# BDNF Val66Met Genetic Polymorphism Results in Poor Recovery Following Repeated Mild Traumatic Brain Injury in a Mouse Model and Treatment With AAV-BDNF Improves Outcomes

**DOI:** 10.3389/fneur.2019.01175

**Published:** 2019-11-07

**Authors:** Anna O. Giarratana, Shavonne Teng, Sahithi Reddi, Cynthia Zheng, Derek Adler, Smita Thakker-Varia, Janet Alder

**Affiliations:** Department of Neuroscience and Cell Biology, Rutgers Robert Wood Johnson Medical School, Piscataway, NJ, United States

**Keywords:** BDNF, lateral fluid percussion, Val66Met, rmTBI, AAV-BDNF

## Abstract

Clinicians have long noticed that some Traumatic Brain Injury (TBI) patients have worse symptoms and take a longer time to recover than others, for reasons unexplained by known factors. Identifying what makes some individuals more susceptible is critical to understanding the underlying mechanisms through which TBI causes deleterious effects. We have sought to determine the effect of a single nucleotide polymorphism (SNP) in Brain-derived neurotrophic factor (BDNF) at amino acid 66 (rs6265) on recovery after TBI. There is controversy from human studies as to whether the BDNF Val66Val or Val66Met allele is the risk factor for worse outcomes after brain trauma. We therefore investigated cellular and behavioral outcomes in genetically engineered mice following repeated mild TBI (rmTBI) using a lateral fluid percussion (LFP) injury model. We found that relative to injured Val66Val carriers, injured Val66Met carriers had a larger inflammation volume and increased levels of neurodegeneration, apoptosis, p-tau, activated microglia, and gliosis in the cortex and/or hippocampus at 1 and/or 21 days post-injury (DPI). We therefore concluded that the Val66Met genetic polymorphism is a risk factor for poor outcomes after rmTBI. In order to determine the mechanism for these differences, we investigated levels of the apoptotic-inducing pro BDNF and survival-inducing mature BDNF isoforms and found that Met carriers had less total BDNF in the cortex and a higher pro/mature ratio of BDNF in the hippocampus. We then developed a personalized approach to treating genetically susceptible individuals by overexpressing wildtype BDNF in injured Val66Met mice using an AAV-BDNF virus. This intervention improved cellular, motor, and cognitive behavior outcomes at 21 DPI and increased levels of mature BDNF and phosphorylation of mature BDNF's receptor trkB. This study lays the groundwork for further investigation into the genetics that play a role in the extent of injury after rmTBI and highlights how personalized therapeutics may be targeted for recovery in susceptible individuals.

## Introduction

Traumatic Brain Injury (TBI) is a serious and potentially life-threatening clinical problem. It occurs when there is a force to the head, which results in a disruption of brain function. In 2013, there were 2.5 million TBIs in the United States, 50,000 of which led to death and 70,000 of which led to permanent neurological damage (1). Based on the neurological symptoms that occur after a TBI, the injury is classified either as mild, moderate, or severe. Mild TBI is the most prevalent form of TBI that occurs in the United States. In particular, athletes and military personnel tend to suffer from mild and repeated traumatic brain injuries (2, 3). While the majority of patients who suffer from mild TBI tend to recover over time, it is estimated that around 15% of patients have symptoms that last longer than 3 months and develop into chronic disabilities (4). This problem is only exacerbated when a person is subjected to repeated mild TBI (rmTBI). It is becoming more evident that some athletes and military personnel who have suffered from mild repeated brain injuries can end up developing neurodegenerative diseases (5). TBI should be thought of as an ongoing disease process, rather than a discrete event. The primary injury occurs as the result of mechanical force to the brain. After the initial insult, a secondary injury process occurs as a result of inflammation and secondary mediators. While some patients who suffer from TBI recover quickly and have no obvious long-term symptoms, other patients undergo a prolonged secondary injury phase and have a much harder time recovering (6, 7). One of the reasons that not all individuals respond similarly to rmTBI may be due to the genetic differences that exist in the population. However, specific genes that may affect outcomes after rmTBI have not been identified.

A critical neuronal gene that contains a single nucleotide polymorphism (SNP) is brain–derived neurotrophic factor (BDNF), a neurotrophin that plays a role in neuronal survival and synaptic plasticity (8–10). BDNF has a SNP site, rs6265, at the 66 amino acid position of the BDNF protein, and results in the wildtype Val being replaced with a Met. The Val66Met genotype has been reported to be associated with poor outcomes in a number of disease states such as major depressive disorder (11), anxiety (12), stroke (13), and Alzheimer's disease (14). However, clinical research in TBI patients is not consistent as to whether the Met or the Val polymorphism confers worse outcomes (15–21). The Val66Met polymorphism results in diminished BDNF in dendrites and reduced amounts of BDNF protein released into the synapse upon stimulation (22). (23). In healthy populations, individuals with the Val66Met polymorphism have been found to have impaired cognitive function (24). It seems to follow logically that the Val66Met polymorphism has been shown to result in worse neurocognitive performance after TBI (21), as well as being a risk factor for TBI in combat forces (18–20). However, some recent studies in humans have shown that counterintuitively, after injury, the Val66Val carriers actually exhibit worse recovery compared to the Val66Met carriers (15–17, 25). Specifically, in long-term studies of combat veterans, carriers of the Val66Met polymorphism recovered their executive function back to baseline levels while the Val66Val carriers did not (15, 17). On the other hand, different studies have found no effect of the polymorphism influencing outcomes after injury (26–28). Thus, the clinical research as to which BDNF allele is the risk factor for outcomes after TBI is not all in agreement, and could benefit from a bottom-up, controlled experimental research study as has been done to examine other disease models where genetic polymorphisms may play a role (29). This study will play an important role in helping to ameliorate some of the controversy that exists about the role of the Val66Met allele after TBI.

The situation is complicated by the fact that the BDNF protein has two important and varied forms, the pro form and the mature form. Mature BDNF binds to its receptor trkB and stimulates neurogenesis and cell survival. ProBDNF protein binds to the p75 receptor and activates the apoptotic cascade (30). We have previously shown that the pro form of BDNF and its signaling pathways are preferentially upregulated after TBI (31). While other groups have shown that the Val66Met genetic polymorphism affects levels of total BDNF in dendrites without altering the relative levels of pro and mature BDNF, the effect that this polymorphism has on levels of pro and mature BDNF after injury and the effect this has on injury outcomes have not been elucidated (32, 33). BDNF also has a third form, the truncated form, whose role is less clear but is thought to be generally beneficial (34).

In this investigation, we studied the effect of the BDNF rs6265 genetic polymorphism on cellular, biochemical, and behavioral changes after repeated mild lateral fluid percussion (LFP) brain injury in mice. The LFP model of TBI is a longstanding method used due its ability to mimic injuries seen in humans, by involving both focal and diffuse components (31, 35–37). Moreover, we focused on repeated mild TBI since it is a common yet understudied form of TBI. Cellular changes were investigated at 1 and 21 DPI, in order to ascertain a more complete picture of the various biological processes that are activated at different time points after injury.

We have shown that after rmTBI, Val66Met mice have increased area of inflammation, cell death, neurodegeneration, p-tau, astrogliosis, and activated microglia at 1 and/or 21 DPI in the cortex and hippocampus compared to Val66Val injured mice. When investigating the relative levels of pro and mature BDNF after injury in these mice, we found that injured Met carriers have less total BDNF in the cortex at 21 DPI, and more pro-BDNF relative to mature BDNF in the hippocampus at 1 DPI compared to injured Val carriers. Finally, we show that when Val66Met carriers are treated with an AAV virus vector to overexpress BDNF, we can rescue the high levels of astrogliosis and activated microglia down to the levels observed in Val66Val injured mice. Treatment with AAV-BDNF also improves learning and memory in Val66Met injured mice in the Morris Water Maze paradigm to the level observed in Val66Val injured mice. To our knowledge, this is the first report showing that there is genotypical susceptibility to poor outcomes after TBI that can be rescued by altering neurotrophic signaling.

## Materials and Methods

### Animals

Adult male and female mice aged 10–12 weeks were used in all studies. BDNF mice were generously provided by Dr. Francis S. Lee of Weil Cornell Medical College (38). The mice were created utilizing a targeting vector with or without the point mutation (G196A) which is regulated by the endogenous mouse BDNF promoter. The colony was maintained by crossing BDNF^*Val*/*Met*^ mice which yield offspring at Mendelian rates. Mice were housed in a 12 h light/dark cycle with food and water available *ad libitum*. All procedures described were performed in accordance to the NIH guidelines and were approved by the Rutgers University Institutional Animal Care and Use Committee (IACUC). A power analysis was used to determine the appropriate sample size for experiments to reach 80% power; for histology the group size *n* = 5–8, for biochemistry the group size *n* = 4–6, and for behavioral tasks *n* = 8–10 were used to reliably detect changes of the magnitude we are examining (α = 0.05) based on the difference seen between experimental groups in our previous publication (31).

### Lateral Fluid Percussion Injury

Lateral fluid percussion injury uses a rapid fluid pulse to cause injury to the brain by the displacement of neural tissue. This process has previously been described in detail (35) but has been modified to create repeated mild injury. Briefly, mice were anesthetized using 4–5% isoflurane in 100% O_2_ and maintained on 2% isoflurane throughout the procedure. They were placed in a stereotaxic frame, and a trephine-guide 3 mm plastic disc was attached with Loctite glue (444 Tak Pak, Henkel Corporation, Rocky Hill, CT) on the skull, halfway between lambda and bregma, laterally on the right hemisphere. A trephine (3 mm outer diameter) was used to perform a craniectomy. A rigid Luer-loc needle hub (3 mm inside diameter) was secured onto the skull over the opening that was made using cyanoacrylate adhesive and dental acrylic (Henry Schein, Dublin, OH). After a 60 min recovery period, the animals were re-anesthetized and connected to the fluid percussion injury device (Custom Design and Fabrication, Virginia Commonwealth University) through the Luer-loc hub. Once the animals regained normal breathing, before sensitivity to stimulation, a ~0.8 ATM pulse (15 ms) was generated through the LFP device to strike the intact dura of the brain. Upon return of righting reflex (<4 min for mild injury) the hub was filled with saline and capped. Forty eight hours from the initial injury, a second injury was given. This occurred again at 96 h from the initial injury. This experimental timeline was chosen based on previous studies which have sought to mimic human repeated mild TBIs in a mouse model which controls for the rodent life span (39–42). After the 3rd injury, the hub and dental acrylic were removed and the scalp incision was closed with 3M Vetbond (Fisher Scientific, Waltham, MA). The animals were individually housed after the injury and returned to normal housing conditions. In order to determine humane endpoints, the mice were monitored twice daily. If signs of pain were detected, the vivarium veterinary staff were contacted, and appropriate analgesics were used immediately. Signs of pain and distress included animals that were no longer able to move to get food or water, or showed signs of pain (ex. hunched posture, inappetence, lethargy, decreased body condition). In order to prevent harm and suffering, we gave a surgical pre-emptive analgesia, in the form of an injection of buprenorphrine (0.1 mg/kg SC). During the surgery, the mice were anesthetized with isoflurane while they were in the stereotaxic apparatus. Adequate anesthetic depth was checked for by a no response to toe pinch before surgery commenced. During the surgery procedure, the anesthetic bupivicaine (0.025%) was applied topically to the skull. In addition, the respiratory rate was monitored throughout the entire surgical procedure and the eyes were protected with lubricant. If necessary, post-op pain medication of Carprofen would be given at 5 mg/kg, SC, once a day and continued if signs of pain were observed (not found to be necessary for any mice in this study). With this repeated, mild level of injury, about 5–10% of animals died after the 3rd injury in the chronic post-traumatic period. In this study, we had a mortality rate of 8.8% with 26/295 deaths. The expectation of this mortality was approved by our institutional IACUC. This is a normal and anticipated feature of the LFP TBI model because it mimics human TBI. Mice that underwent the surgical procedure but not the injury were used as sham controls. Assignment of the mice to the LFP or sham group was randomized.

### MRI Imaging

Magnetic resonance imaging (MRI) was done on a cohort of mice in order to assess the volume of inflammation as determined by increased relative intensity (ROI). The scans were done utilizing a fast spin echo sequence with a mouse brain coil. Scans were done in the axial position at 1, 7, and 21 days after the final injury. Inflammation was determined through an auto-thresholding to analyze higher intensity areas relative to regular brain tissue. All brains were reviewed with same intensity search and normalized using the Image Scale Factor in the VivoQuant Analysis Software. The region of interest was determined by analyzing areas of increased intensity within specific coordinates in the damaged location of the brain and analyzed blinded to condition. Scans were done at the Rutgers University Molecular Imaging Center with the center's M2 Compact High-Performance MRI (1T).

### Immunohistochemistry

To collect tissue for immunohistochemistry, a second cohort of mice were perfused with 0.9% saline, followed by 4% paraformaldehyde at 1 and 21 days after the final injury. After perfusion, the brains were cryoprotected with 30% sucrose for at least 3 days. Sectioning was done in 20 μm thick slices, in a 1:10 series throughout the length of the hippocampus, incorporating the area around the site of injury in the cortex. To measure apoptotic cell death, sections were pretreated with 0.01 M Citrate buffer at 90°C. Anti-cleaved caspase-3 (1:1,000, 9,661, Cell Signaling, Danvers, ME) was then applied overnight, followed by Alexa Fluor 594 goat anti-rabbit (1:1,000, Invitrogen, Waltham, MA). To measure astrogliosis, Glial Fibrillary Acidic Protein (GFAP) antibody was applied overnight (1:500, MAB3402, Millipore, Billerica, MA), followed by Alexa Fluor goat anti-mouse 488 (1:500, Invitrogen, Waltham, MA). To measure neuronal degeneration, sections were first treated with 1% NaOH and 0.06% KMnO_4_, then 0.0005% Fluoro-Jade C (AG325, Millipore, Burlington MA)/0.0001% DAPI (D9564, Sigma, St. Louis, MO) was applied for 20 min. To measure microglial activation, IBA1 antibody was applied overnight (1:10,000, 019-19741, Wako Labs, Richmond, VA), followed by Alexa Flour goat anti-rabbit 488 (1:1,000, Invitrogen, Waltham, MA). To measure levels of phosphorylated tau, AT8 antibody was applied overnight (1:500, MN1020, Pierce Antibodies, Waltham, MA), followed by Alexa Fluor goat anti-mouse 488 (1:1,000, Invitrogen, Waltham, MA). All slides were incubated in 4',6-diamidino-2-phenylindole (DAPI) (1:1,000 DAPI in PBS, Sigma, St. Louis, MO). Slides were mounted in Fluoromount-G (Southern Biotech, Birmingham, AL), except for the Flouro-Jade C slides which were mounted in DPX Mountant (44581, Sigma, St. Louis, MO). Visualization of the fluorescent stains was done using a Leica microscope (Model DMIRB, Leica Microsystems, Buffalo Grove, IL). Five to eight animals per time point and treatment were analyzed. Sectioning of tissue was done using a Cryostat (Leica) and collected coronally in 1:10 series throughout the length of the hippocampus. For each biological replicate, the collected sections of brain were counted and the average number of cells per section was calculated. Positive cells were counted in the hemisphere ipsilateral to the injury. In the cortex, for each section, six fields of 40X view (starting at the dorsal midline and moving laterally for three fields of vision, and then the three fields of vision just ventral to the first three) were counted. In the hippocampus, the dentate gyrus as well as the CA1-CA3 were used for quantification of cells. Analysis was performed blind to experimental group and genotype.

### Vestibular Rotarod Test

In order to study the vestibular motor abilities of the mice after LFP, the rotarod test was conducted as part of a behavioral battery on a third cohort of mice (Supplemental Figure 3). The rotarod test utilized a 36-mm outer diameter, rotating rod whose velocity increased from 4 to 40 rpm over a maximum 180 s interval. Balance and motor function were measured using the latency to fall. Each trial ended when the animal fell off the rotarod. Eight to ten mice per genotype and condition were used. Acclimation and baseline analysis were done 1 day prior to the first injury, using three trials separated by a 1-h inter-trial rest phase. At 1, 7, and 21 days after the last injury, each mouse underwent three trials separated by a 1-h inter-trial rest phase. The same mice were used for each time point and analyzed blinded to condition. The average latency to fall was compared between injured and sham groups.

### Balance Beam Test

In order to study fine motor function, the balance beam test was conducted. The beam apparatus consists of a one meter long flat beam with a width of 20 mm, raised 30 cm above the table surface. A black box was placed at one end of the beam as the finish point. The mice were pretested on the beam apparatus for 4 days before the test day for training and baseline measurements. On test day, the mice were observed crossing the beam while the number of paw faults, falls, and relative time to cross were recorded manually. Mice were tested at 7 and 21 DPI, and the same mice were used for each time point. Eight to ten mice per genotype and condition were used. Values were imputed into a predetermined scale to evaluate outcomes with weighted values for the different traits analyzed in order to account for the severity of injury indicated by each. A score of 1 was standard for all mice, the number of falls was added after being multiplied by 2, the number of foot faults were added, and if the mouse crossed the beam in under 5 s, a score of 1 was removed from the final score. Analysis was done blinded to condition.

### Morris Water Maze Test

In order to study spatial memory, the Morris water maze test was done. Mice were acclimated to the paradigm and tested for baseline response using a visible platform test 1 day prior to the start of the injury paradigm. The animals were placed in a circular pool (1 m diameter) filled with opaque water containing non-toxic white paint and a clear escape platform marked by a visible rod. To assess learning, the mice were tested using a hidden platform fixed in the northwest quadrant starting 1 day after the last injury. Testing was conducted with four trials a day for 6 days in a row. On the seventh day, a probe test was completed to test memory, where the hidden platform was removed and the time spent exploring the northwest quadrant was recorded. Black and white distal extra-maze cues were positioned on the walls of the room and geometric shaped proximal extra-maze cues were positioned above the walls of the maze. The mice were placed in pseudo-randomly varied quadrants throughout testing, and the time to locate the platform was recorded. Trials were run until the mouse found the platform or was placed there after the maximum trial time of 60 s. At the conclusion of the trial, the mouse was allowed to remain on the hidden platform for 15 s to consolidate learning, followed by removal from the pool and placement onto a heating pad for 10 min. Eight to ten mice per group and condition were used. Data was analyzed blinded to condition. Data was recorded using a video-tracking system (EthoVision XT; Noldus Information Technology, Leesburg, VA).

### Western Blot Analysis

The cortex and hippocampus on the ipsilateral side to the injury site were collected from mice at 1 and 21 dpi and flash frozen. Four mice per group and condition were analyzed at each timepoint. Tissue lysates were prepared using T-PER with protease inhibitors and EDTA (Pierce, Rockford, IL). Samples were homogenized for 30 s and then centrifuged for 10 min. The protein content of the supernatant was determined using the bicinchoninic acid (BCA) Protein Assay Reagent Kit (Pierce, Rockford, IL). Equal amounts of protein were loaded onto Bis Tris Gels (Invitrogen, Grand Island, NY). The proteins were transferred onto polyvinylidene difluoride (PVDF)-filter Immobilon-P transfer membranes (Millipore, Billerica, MA). Following blocking in 5% BSA + 5% normal donkey serum overnight at 4°C, the primary antibody was applied overnight at 4°C. Forty microgram of protein was run on a 12% Bis Tris gel and probed for pro and mature BDNF (1:500 BDNF Icosagen, San Francisco, CA). Glyceraldehyde 3-phosphate dehydrogenase (GAPDH) antibody (1:1,000, Biodesign, Saco, ME) was used as a loading control. Forty microgram of protein was run on a 4–12% Bis Tris gel and probed for p-trkB (1:1,000, EMD Millipore, Burlington, Ma). trkB was used as a loading control (1:1,000, Sigma-Aldrich, St. Louis, MO). Secondary anti-mouse or anti-rabbit horseradish peroxidase (HRP)-conjugated IgG antibodies were used (1:5,000, GE Healthcare, South Plainfield, NJ). GAPDH protein was visualized by chemiluminescence using the Enhanced Chemiluminescence (ECL) detection kit (Perkin Elmer, Waltham, MA) and all others were visualized using the SuperSignal West Femto Maximum Sensitivity Substance (ThermoFisher Scientific, Waltham MA). Levels of the immunopositive bands were quantified densitometrically using Quantity One version 4.2.1 software on a GelDoc 2000 (Bio-Rad, Hercules, CA). All data is normalized to the sample's own GAPDH and expressed as a fold change relative to the average of the genotype matched sham controls.

### BDNF Viral Infusion

An AAV9-CMV-GFP-2A-mouseBDNF construct expressing the wildtype 66Val form of BDNF at a titer of 4.5 × 10^13^ viral genomes/μL was purchased from Vector BioLabs (Malvern, PA) and dose selection was done in conjunction with the company. Mice were anesthetized using 4–5% isoflurane in 100% O_2_ and maintained on 2% isoflurane throughout the procedure. They were placed in a stereotaxic frame, and a 32 G Hamilton Neuro syringe was used to deliver a volume of 0.75 μL at a speed of 0.25 μL/min into both the ipsilateral cortex (AP −1.9 mm, ML, −1.5 mm, DV −1.5 mm) and hippocampus (AP −1.9 mm, ML, −1.5 mm, DV −2.5 mm) of animals 5 min after the final LFP injury. The needle was left in place after injection for 5 min to allow for completion infusion of the drug. The control group received the same injection protocol with a control AAV-CMV-GFP construct 4.5 × 10^13^ viral genomes/μL purchased from Vector BioLabs. Analysis was done as is standard in the field, assuming dose dependent GFP expression (43, 44).

### Statistical Analysis

StatPlus software was used for all data analysis. Groups were compared using Student's two-tailed *t*-test or one-way ANOVA followed by Fisher's PLSD *post-hoc* analysis. *p* < 0.05 is considered statistically significant. Statistical results are presented in the figures and legends and the *p*-values are provided in Supplemental Figure 4.

## Results

### Val66Met Injured Mice Have a Larger Volume of Inflammation Compared to Val66Val Injured Mice at 21 DPI Following rmTBI

**Figure 1:**
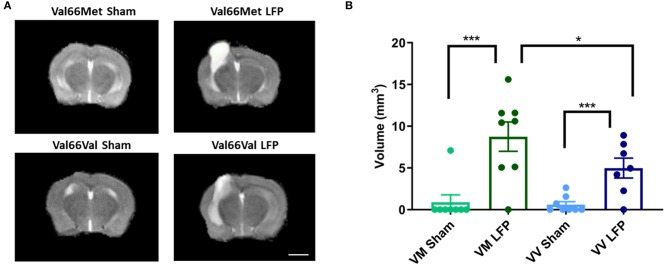
Val66Met injured mice have greater volume of inflammation than Val66Val injured mice. **(A)** Representative MRI images of mice subjected to LFP and sham at 21 days after the final injury. **(B)** Quantification of volume of inflammation in different genotypes, 21 DPI as determined by assessment of hyperintensity ROI. ^*^p < 0.05, ^***^p < 0.001, ANOVA Fisher's PLSD *post-hoc* test relative to indicated groups, *n* =7–8. Scale bar = 3 mm.

Throughout this report, we have compared differences in assay outcomes between the injured and sham groups, as well as between the two injured genotypes Val66Met and Val66Val. To investigate the role that genotype plays on the volume of inflammation after our repeated mild LFP model, we used a 1T MRI to scan the brains of the mice at 1, 7, and 21 DPI. Utilizing T2 fast spin echo sequence imaging scans, we found that by 21 DPI, edema at the site of the craniectomy returned to pre-injury levels in sham condition mice when the same mice were imaged over multiple time points, while in the injured mice there was still edema and swelling present. Some of this edema extended through the hole in the skull left by the craniectomy resulting in extra-axial hyperintensity as part of the injury area (Supplemental Figure 1). Due to the persistence of edema, we selected the 21 DPI time point to assess the effect of the BDNF SNP on volume of inflammation. At 21 DPI, there was a significant difference between the volume of inflammation in sham and injured mice as determined from measuring the hyperintensity volume of T2 MRI scans. Particularly of interest, we saw that Val66Met injured mice had a significantly larger volume of inflammation compared to Val66Val injured mice (Figure 1). These data suggest that there are differences in the level of injury occurring between these two genotypes at 21 DPI as seen through the increased edema. However, it is not known which processes are affecting this difference.

### Activated Iba1+ Cells Are Increased in Val66Met Injured Mice Compared to Val66Val Injured Mice, While Non-activated Microglia Are Consistent Across All Groups

**Figure 2:**
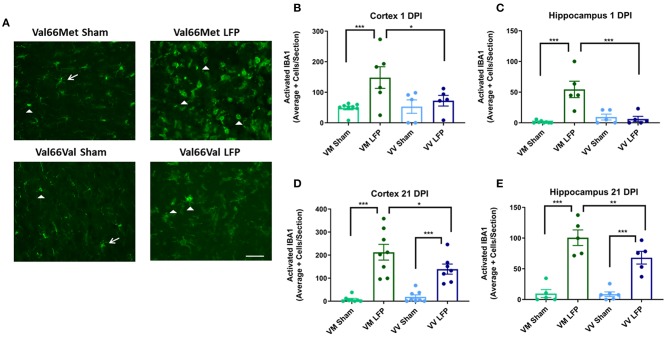
Repeated mild LFP injury causes an increase in ionized calcium binding adaptor molecule 1 (IBA1) positive cells in the brains of injured Val66Met mice compared to injured Val66Val mice at 1 DPI. **(A)** Representative images of cortical sections at 1 DPI stained with IBA1. White arrows indicate resting microglia, white arrowheads indicate activated microglia. Scale bars = 100 μm. **(B–E)** Quantification of the average number of IBA1+ positive cells, broken down into activated and resting categories by morphology, per cortex and hippocampus ± SEM. ^*^p < 0.05, ^**^p < 0.01, ^***^p < 0.001, ANOVA Fisher's PLSD *post-hoc* test relative to indicated groups, *n* =5–8.

To study the underlying cellular changes that contribute to the genetic difference in volume of inflammation, we conducted immunohistochemical staining at both 1 and 21 DPI to assess the immediate and longer lasting effects of rmTBI on various cellular processes. As our LFP injury paradigm includes both focal and distal components, we analyzed the ipsilateral cortex to gain an appreciation of the focal components of injury as well as its possible effects on sensorimotor function. We also analyzed the ipsilateral hippocampus to investigate the slightly distal effects of injury and to gain insights into the possible effect on cognitive function. The repeated mild LFP mouse model was chosen because it is able to accurately mimic the injuries that are seen after human repeated mild TBI; there is an acute injury at the point of contact, as well as diffuse injury in other brain areas (45). As expected by this model, we see mild signs of injury on the contralateral side of the brain demonstrated by microglial staining (Supplemental Figure 5). Therefore, instead of using the contralateral cortex and hippocampus as controls, we chose to use mice that have undergone craniectomy surgery but no injury (sham) and analyzed the ipsilateral hemisphere of those mice. First, to examine the effect that repeated mild TBI has on the neuroimmune system, we analyzed activated microglia. Activated microglia are an important part of the secondary injury process, have been shown to persist for years after the initial injury, and contribute to long-term neurological dysfunction (46). We used IBA1 as a marker for microglia and we utilized morphology to distinguish activated microglia from non-activated microglia. Non-activated microglia were identified by their ramified appearance, while activated microglia were in either the reactive bushy state or the phagocytic ameboid shape (47). We found that at 1 DPI Val66Met injured mice had significantly more activated microglia than their sham controls in both the ipsilateral cortex and the hippocampus. Notably, we found that at 1 DPI Val66Met injured mice had significantly more activated microglia than the Val66Val injured mice in the ipsilateral cortex or hippocampus (Figures 2B,D). These data indicate that Val66Met injured mice have earlier microglial activation than the Val66Val injured mice. By 21 DPI, the Val66Met and Val66Val injured mice both had significantly more activated microglia than their sham controls. Again, we observe a significant difference in the levels of activated microglia in the Val66Met injured mice compared to the Val66Val injured mice at 21 DPI in both the ipsilateral cortex and hippocampus (Figures 2C,E). These data suggest that Val66Met injured mice respond to repeated mild injury by activating microglia earlier than Val66Val injured mice, and this increased activation is sustained through 21 DPI.

### Levels of Activated Caspase-3+ Cells Are Higher in Val66Met Injured Mice Compared to Val66Val Injured Mice at 1 DPI, but Not at 21 DPI

**Figure 3:**
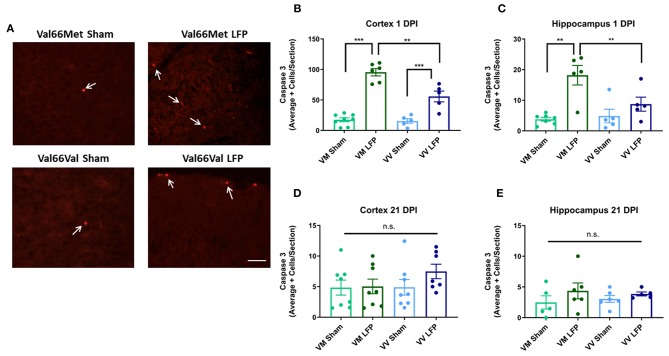
Repeated mild LFP injury causes an increase activated caspase-3 positive cells in brains of injured Val66Met mice compared to injured Val66Val mice at 1 DPI. **(A)** Representative images of cortical sections at 1 DPI stained with activated caspase-3 (indicated by arrows). Scale bars = 100 μm. **(B–E)** Quantification of the average number of activated caspase-3 positive cells per cortical or hippocampal section ± SEM. ^**^p < 0.01, ^***^p < 0.001, ANOVA Fisher's PLSD *post-hoc* test relative to indicated groups, *n* = 5–8.

Since increased levels of neuronal cell death are common following injury (31, 48), we used activated caspase-3 to assess levels of apoptosis. We found that at 1 DPI, both Val66Met and Val66Val injured mice have a significant increase in the number of activated caspase-3 positive cells relative to their sham controls indicating that apoptosis was at higher levels at 1 DPI. Importantly, Val66Met injured mice had a significantly higher number of activated caspase-3 positive cells relative to the Val66Val injured mice, in both the ipsilateral cortex and hippocampus (Figures 3A–C) suggesting that Val66Met mice have more cell death after rmTBI than Val66Val mice. However, by 21 DPI levels of cell death had decreased so that there was no detectable difference between the injured mice and their sham controls (Figures 3D,E). This is similar to what we have previously seen after single moderate injury by 21 DPI (31). These data suggest that while there are initial genotypic differences in apoptotic cell death after injury with Val66Met exhibiting worse outcomes than Val66Val that these differences are resolved by 21 DPI.

### Levels of FluorojadeC+ Cells Are Increased in Val66Met Injured Mice Compared to Val66Val Injured Mice at 1 DPI and 21 DPI

**Figure 4:**
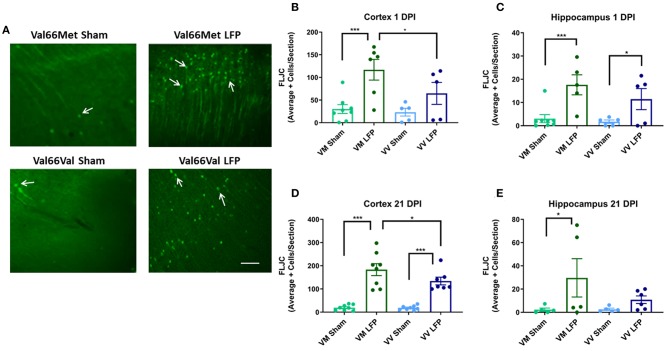
Repeated mild LFP injury causes an increase in Fluoro-Jade C (FLJC) positive cells in the brains of injured Val66Met mice compared to injured Val66Val mice at 1 and 21 DPI. **(A)** Representative images of cortical sections at 1 DPI stained with FLJC (indicated by arrows). Scale bars = 100 μm. **(B–E)** Quantification of the average number of FLJC positive cells per cortex or hippocampus ± SEM. ^*^p < 0.05, ^***^p < 0.001, ANOVA Fisher's PLSD *post-hoc* test relative to indicated groups, *n* = 5–8.

Increased levels of neurodegeneration are common sequelae following injury to the brain (31, 42). We used Fluorojade C (FLJC), a marker for neurodegeneration (49), in order to ascertain the level of neurodegeneration in the ipsilateral cortex and hippocampus. We found that at 1 DPI, Val66Met injured mice had significantly more FLJC positive cells in both the ipsilateral cortex and hippocampus relative to their sham controls (Figures 4B,C). At this timepoint, Val66Val injured mice did not significantly differ from their sham controls in numbers of FLJC positive cells in the ipsilateral cortex, although Val66Val mice did differ from their sham controls in the hippocampus. Importantly, the Val66Met injured mice had more FLJC positive cells than the Val66Val injured mice in the ipsilateral cortex at 1 DPI, indicating that the Val66Met injured mice have more neurodegeneration at this early timepoint. By 21 DPI, both the Val66Met injured mice and the Val66Val injured mice had significantly more FLJC positive cells than their sham controls in the ipsilateral cortex, and notably, the Val66Met injured mice had significantly more FLJC positive cells than the Val66Val injured mice at this time point as well (Figure 4D). In the hippocampus at 21 DPI there was still a significant difference between the injured Val66Met mice and their sham controls, but no detectable difference between the Val66Val mice and their sham controls (Figure 4E). There was also still no detectable difference between the Val66Met and Val66Val injured mice at 21 DPI in the hippocampus. These data suggest that the neurodegeneration process begins as early as 1 DPI and that increased levels of neurodegeneration in response to injury may be sustained until at least 21 DPI in the Val66Met injured mice.

### Number of Phosphorylated tau+ Cells Are Increased in Val66Met Injured Mice Compared to Val66Val Injured Mice at 1 and 21 DPI

**Figure 5:**
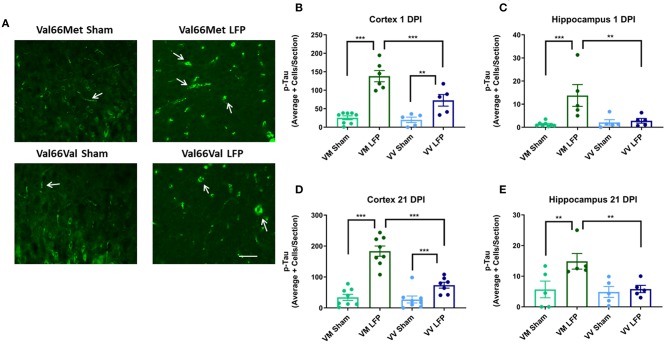
Repeated mild LFP injury causes an increase in phosphorylated tau (p-tau) in the brains of injured Val66Met mice compared to injured Val66Val mice at 1 and 21 DPI. **(A)** Representative images of cortical sections at 1 DPI stained with activated AT8 (indicated by arrows). Scale bars = 100 μm. **(B–E)** Quantification of the average number of p-tau positive cells per cortex or hippocampus ± SEM. ^**^p < 0.01, ^***^p < 0.001, ANOVA Fisher's PLSD *post-hoc* test relative to indicated groups, *n* = 5–8.

It has previously been shown that phosphorylated tau can contribute to long-term pathologies in the brain (50). After injury, there are frequently higher levels of phosphorylated tau at the site of injury as well as in distally affected brain areas (51). We investigated the levels of phosphorylated tau at 1 and 21 DPI in both the ipsilateral cortex and hippocampus. We found that there was a significant increase in levels of phosphorylated tau in both Val66Met and Val66Val injured mice compared to their sham controls in the ipsilateral cortex at both 1 and 21 DPI (Figures 5B,C). Of note, the Val66Met injured mice had significantly more phosphorylated tau compared to Val66Val injured mice, seen at 1 DPI and sustained through to 21 DPI. This suggests that Val66Met injured mice may have an exacerbated phosphorylated tau reaction after injury that begins at 1 DPI and is sustained until 21 DPI. In the hippocampus, we found that the Val66Met injured mice had significantly more phosphorylated tau than their sham controls at both 1 and 21 DPI, while the Val66Val injured mice did not differ from their sham controls (Figures 5D,E). These data suggest that after repeated mild injury, Val66Met mice are uniquely susceptible to mechanisms that result in increased levels of phosphorylated tau in the hippocampus.

### The Number of GFAP+ Cells Are Increased in the Ipsilateral Cortex in Val66Met Injured Mice Compared to Val66Val Injured Mice by 21 DPI, but Not at 1 DPI

**Figure 6:**
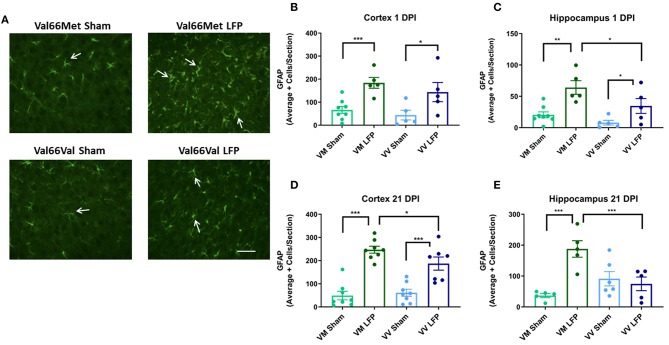
Repeated mild LFP injury causes an increase in glial fibrillary acidic protein (GFAP) positive cells in the brains of injured Val66Met mice compared to injured Val66Val mice at 1 DPI. **(A)** Representative images of cortical sections at 1 DPI stained with glial fibrillary acidic protein (indicated by arrows). Scale bars = 100 μm. **(B–E)** Quantification of the average number of GFAP positive cells per cortex or hippocampus ± SEM. ^*^p < 0.05, ^**^p < 0.01, ^***^p < 0.001, ANOVA Fisher's PLSD *post-hoc* test relative to indicated groups, *n* = 5–8.

It is well documented that after injury there is proliferation of glia (31, 42) that can contribute to the formation of glial scarring, inhibition the ability of neurons to regenerate, and prevention of the injured brain from recovering normal morphology and function (52). Using glial fibrillary acidic protein (GFAP), a marker for activated astrocytes, we quantified the level of gliosis after injury. We found that at 1 DPI, both Val66Met and Val66Val injured mice had more astrogliosis than their sham controls in the cortex and hippocampus (Figures 6B,C). Interestingly, we found that the Val66Met injured mice had more astrogliosis than Val66Val injured mice in the hippocampus at this 1 DPI, but not in the cortex, suggesting that the differential genotypic effects on astrocytes are more evident earlier in the time course in the more distal hippocampal region. By 21 DPI, both the Val66Met and Val66Val injured mice had significantly more astrogliosis than their sham controls in the cortex, although by this timepoint only the Val66Met injured mice remained detectably different from their sham controls in the hippocampus (Figures 6D,E). In analyzing genotypic differences, we found that the injured Val66Met mice also had increased astrogliosis relative to Val66Val injured mice in the ipsilateral cortex and hippocampus at 21 DPI. These data suggest that activation of astrocytes after injury is variable based on genotype, and that Val66Met mice have more injury-related cellular changes compared to Val66Val mice.

### Injured Met Carriers Have Less Total BDNF in the Cortex at 21 DPI, and More pro-BDNF/mature BDNF in the Hippocampus at 1 DPI Compared to Injured Val Carriers

**Figure 7:**
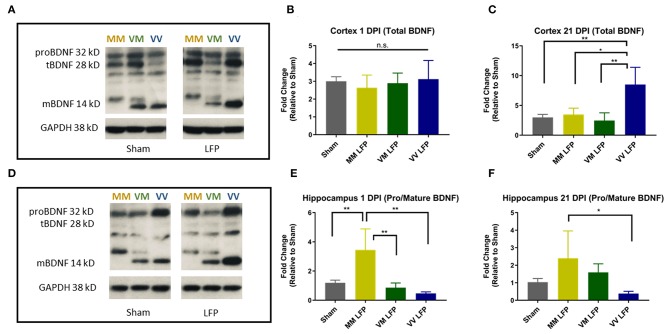
Repeated mild LFP injury causes an increase in proBDNF/mature BDNF expression in Met carriers compared to Val carriers in the hippocampus at 1 and 21 DPI. **(A,D)** Representative Western Blot showing pro and mature BDNF expression in the hippocampus after injury. Each lane represents one animal. **(B,C)** Quantification of protein levels in the cortex at 1 DPI and 21 DPI and **(E–F)** hippocampus at 1 DPI and 21 DPI. All data is first normalized to GAPDH to control for protein loading and then expressed as a fold change relative to the average ± SEM of the time matched sham controls which are represented as a single bar in the graph. ^*^p ≤ 0.05, ^**^p < 0.01, ANOVA Fisher's PLSD *post-hoc* test relative to indicated groups, *n* = 4.

To examine a possible mechanism underlying the differences in genotypic response to rmTBI, we performed biochemical studies looking at levels of pro and mature BDNF. Previous studies have shown that in naïve mice, Val66Met and Met66Met mice have comparable levels of BDNF compared to their Val66Val counterparts, although they have less BDNF protein released from the dendrites (33). However, to our knowledge no one has assessed the levels of pro and mature BDNF after repeated mild TBI. In order to determine the effect of the Met allele on BDNF levels after injury and whether the Met allele functions in a dose dependent manner, we used Western Blot analysis in Val66Val, Val66Met, and Met66Met mice. The Met66Met group was added to this assay in order to get a more thorough understanding of the effect that the Met allele has on BDNF protein levels, although the comparison of the Met66Met group was not the focus of our study in other assays. We found that injured Met carriers did indeed have less total BDNF in the cortex at 21 DPI compared to injured Val carriers (Figures 7A–C). To quantify the amounts of pro and mature BDNF, we analyzed these isoforms separately but combined the mature bands at 14 and 16 kD to account for the differences in molecular weight caused by the Met-His tag on the BDNF transgenic gene. We found that at 1 and 21 DPI, injured Met carriers had more pro-BDNF/mature BDNF than Val carriers (Figures 7D–F). These data suggest that there is differential genotypic response to injury by demonstrating a biochemical alteration in the two genotypes and highlight a potential pathway to target for therapies.

### Administration of AAV-BDNF to Val66Met Injured Mice Reduces the Level of Astrogliosis and Activated Microglia at 21 DPI to the Levels Seen in Val66Val Injured Mice

Analysis of our data suggests that Val66Met mice have more injury-related cellular responses after repeated mild LFP injury than their sham controls and Val66Val injured mice. Our Western Blot data show that there may be alterations in BDNF levels that are affecting outcomes after injury. Therefore, we injected an AAV-BDNF expressing vector, containing the wildtype 66Val form, immediately after the third injury to increase levels of BDNF in Val66Met injured mice and examined cellular and behavioral outcomes, using injections of AAV-GFP as the control. We previously found differences in both cellular and behavioral outcomes between injured and sham mice using our rmTBI model, but no differences between the Val66Met and Val66Val sham groups (Figures 2–6). Therefore, in our rescue study, we decided to focus on the differences we saw between the injured groups and the effect of the treatment on those groups.

**Figure 8:**
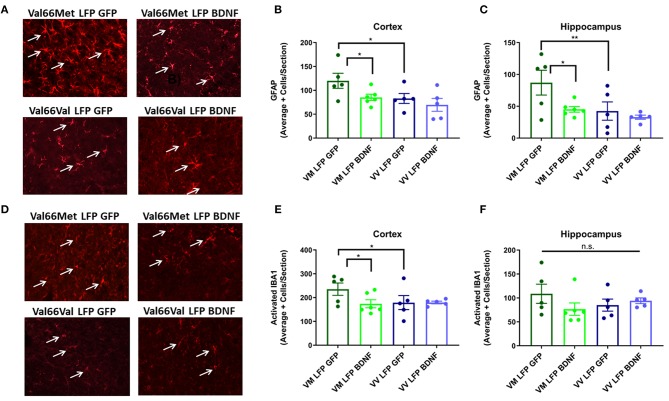
Treatment of injured Val66Met with AAV-BDNF after repeated mild LFP injury causes a decrease in astrogliosis back to injured Val66Val levels in the cortex and hippocampus and activated microglia in the cortex at 21 DPI. **(A)** Representative images of cortical sections at 21 DPI stained with GFAP (indicated by arrows). Scale bars = 100 μm. **(B)** Quantification of the average number of GFAP positive cells per cortex and **(C)** hippocampus ± SEM. **(D)** Representative images of cortical sections at 21 DPI stained with IBA1 (indicated by arrows). Scale bars = 100 μm. **(E)** Quantification of the average number of activated IBA1 positive cells per cortex and **(F)** hippocampus ± SEM. ^*^p ≤ 0.05, ^**^p < 0.01, ANOVA Fisher's PLSD *post-hoc* test relative to indicated groups, *n* = 5–6.

We chose to analyze astrogliosis and activated microglia at the 21 DPI timepoint since we previously found significant differences between the two injured genotypes in these markers and this longer time point allows for expression of the AAV-BDNF throughout the cortex and hippocampus on the ipsilateral side with minimal expression on the contralateral side (Supplemental Figure 2). Val66Met injured mice treated with the control AAV-GFP had significantly higher levels of GFAP+ cells in the cortex and hippocampus and activated IBA1+ cells in the cortex relative to the Val66Met injured mice treated with the AAV-BDNF and the Val66Val injured mice treated with either AAV-GFP or AAV-BDNF at 21 DPI. The AAV-BDNF treated Val66Met mice had levels of astrogliosis and activated microglia that were similar to levels seen in the Val66Val injured mice, both those treated with the control AAV-GFP and AAV-BDNF (Figures 8A–F). These data show that treatment of injured Val66Met mice with AAV-BDNF can decrease astrogliosis and activated microglia relative to injured Val66Met mice treated with control AAV-GFP, suggesting that the AAV-BDNF treatment is able to reduce inflammation after injury.

### Treatment of Val66Met Injured Mice With AAV-BDNF Improves Learning at 16 and 17 DPI, but Not Memory at 21 DPI, Back to the Levels Seen in Val66Val Injured Mice

**Figure 9:**
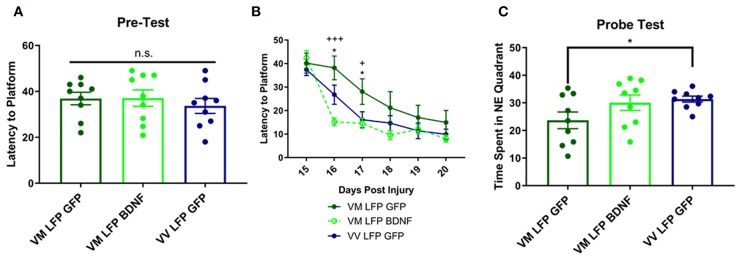
Repeated mild LFP injury causes worse learning and memory in Val66Met injured mice relative to Val66Val injured mice. Treatment with AAV-BDNF in injured Val66Met mice improves learning, but not memory. **(A)** Average latency to platform in pretest phase ± SEM. **(B)** Average latency to platform ± SEM 15–20 DPI. **(C)** Average time spent in the target quadrant in the probe test ± SEM at 21 DPI. ^*^p < 0.05 in Val66Met LFP Control relative to Val66Val LFP Control, ^+^*p* < 0.05, ^+++^*p* < 0.001 in Val66Met LFP Control relative to Val66Met LFP BDNF. ANOVA Fisher's PLSD *post-hoc* test relative to indicated groups, *n* = 9.

We next examined the effect that our injury paradigm has on cognitive function using the Morris Water Maze to study spatial learning and memory. Previous studies have shown that in naïve mice, there is no difference in learning and memory between Val66Val and Val66Met mice (53). Accordingly, we found no difference between groups in the pre-test done before the injury protocol (Figure 9A). However, it has been established that brain injury can have detrimental effects on cognition, especially in animals that have defects in the hippocampus (31, 54). Here, we found that Val66Met injured mice treated with AAV-GFP had a longer latency to find the hidden platform than Val66Val injured mice treated with AAV-GFP at 16 and 17 DPI. Importantly, when the Val66Met injured mice were treated with AAV-BDNF they found the hidden platform significantly faster than the Val66Met injured mice treated with the control AAV-GFP at 16 and 17 DPI, indicating that overexpression of BDNF was able to improve spatial learning at these timepoints (Figure 9B).

In the Probe Test at 21 DPI, we found that the control AAV-GFP Val66Met injured mice spent significantly less time in the target NE quadrant than control AAV-GFP Val66Val injured mice, indicating impaired spatial memory. However, the AAV-BDNF Val66Met injured mice did not spend significantly more time in the target NE quadrant than the AAV-GFP Val66Met injured mice, indicating that treatment with overexpression of BDNF did not improve spatial memory in these mice (Figure 9C). These data suggest that in addition to improving cellular differences seen after injury, that treatment with AAV-BDNF is able to rescue functional cognitive deficits as well.

### Treatment of Val66Met Injured Mice With AAV-BDNF Does Not Improve Motor Function at 14 and 21 DPI Back to the Levels Seen in Val66Val Injured Mice

**Figure 10:**
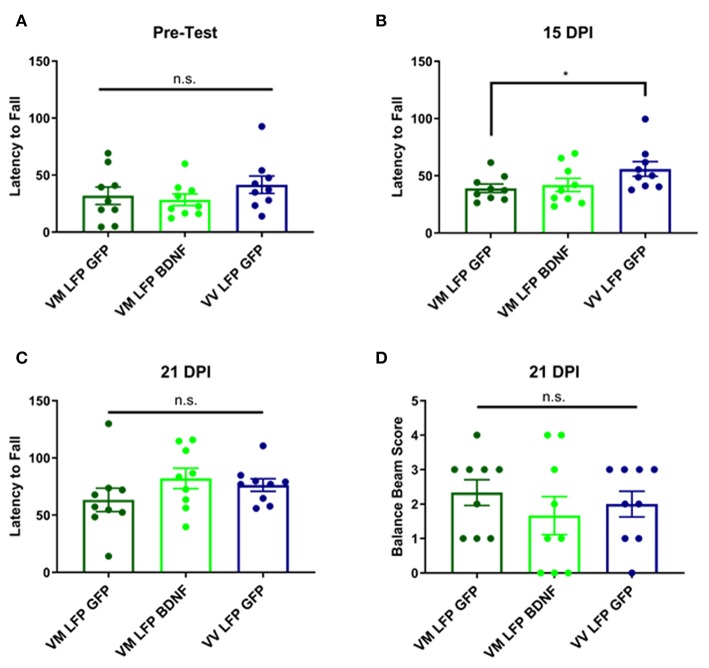
Repeated mild LFP injury causes a deficit in vestibular motor function at 14 days in injured Val66Met mice relative to injured Val66Val. Treatment with AAV-BDNF does not significantly improve this deficit. **(A)** Quantification of latency to fall in the rotarod pretest assay ± SEM **(B)** Quantification of the latency to fall in the rotarod assay ± SEM at 14 DPI and **(C)** 21 DPI. **(D)** Quantification of balance beam score ± SEM at 21 DPI. ^*^p < 0.05, ANOVA Fisher's PLSD *post-hoc* test relative to indicated groups, *n* = 9.

It has previously been shown that following brain injury, there can be deficits in motor ability (54, 55). This is particularly true with our model of LFP, due to the damage that is done at the site of injury to the sensorimotor cortex (56). In order to measure motor ability, we used the rotarod test for vestibular motor function and proprioception and the balance beam test for more subtle differences in motor skills and balance. For the rotarod test, we conducted a pre-test to train the mice on the test, since performance will increase with practice, and to determine if there were any underlying genotypic differences in the mice. We found no differences in the pre-test (Figure 10A) and found that at 15 DPI AAV-GFP treated Val66Met injured mice had a shorter latency to fall compared to AAV-GFP Val66Val injured mice, indicating impaired vestibular motor ability in the Val66Met mice after injury. However, treatment with AAV-BDNF did not shorten the latency to fall in the injured Val66Met mice (Figure 10B). At 21 DPI, we did not find any differences across groups in latency to fall in the rotarod test, indicating that the vulnerable Val66Met injured mice had endogenously improved vestibular motor ability back to levels comparable with Val66Val injured mice (Figure 10C).

For the balance beam test at 21 DPI, we did not observe any difference between the injured Val66Met control AAV-GFP mice and either their Val66Met AAV-BDNF treated counterparts or the Val66Val control AAV-GFP injured mice (Figure 10D). These data suggest that while there may be differences in motor ability at earlier timepoints after injury, by 21 DPI Val66Met control AAV-GFP mice that are acutely vulnerable have recovered back to levels comparable with the less vulnerable Val66Val control AAV-GFP mice. Based on these results, as well as an analysis of swim speed in the MWM, we have concluded that there is no locomotor deficit evident at 21 DPI. Therefore, unlike our single moderate LFP paradigm that creates a significant long-term motor deficit (31), our repeated mild LFP appears to generate more subtle short-term motor deficits.

### Treatment of Val66Met Injured Mice With AAV-BDNF Increases Levels of Total and Mature BDNF as Well as p-trkB in the Cortex and the Hippocampus Relative to Val66Met Injured Mice Treated With Control AAV-GFP

**Figure 11:**
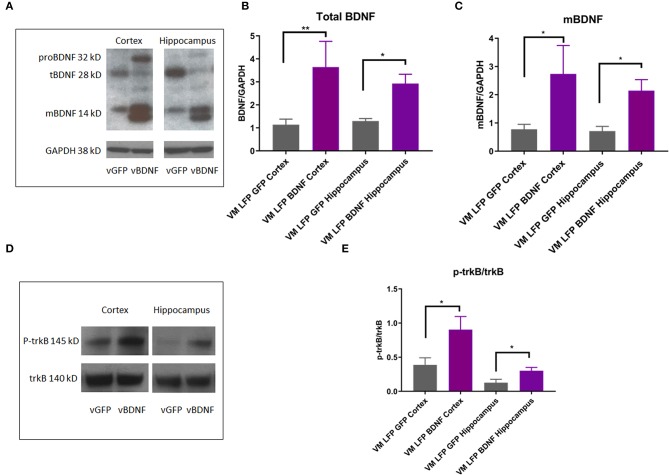
AAV-BDNF injection causes an increase in total BDNF, mature BDNF, and p-trkB expression relative to control AAV-GFP injection in the cortex and hippocampus at 21 DPI in injured Val66Met mice. **(A)** Representative Western Blot showing BDNF expression in the cortex and hippocampus after injury. Each lane represents one animal. **(B)** Quantification of total BDNF protein levels ± SEM at 21 DPI in the cortex and hippocampus. **(C)** Quantification of mature BDNF protein levels ± SEM at 21 DPI in the cortex and hippocampus. All data is first normalized to GAPDH to control for protein loading ± SEM, *n* = 6. **(D)** Representative Western Blot showing p-trkB expression in the cortex and hippocampus after injury. Each lane represents one animal. **(E)** Quantification of p-trkB protein levels at 21 DPI in the cortex and hippocampus. All data is first normalized to trkB to control for protein loading ± SEM, *n* = 4. ^*^p < 0.05, ^**^p < 0.01, ANOVA Fisher's PLSD *post-hoc* test relative to indicated groups.

In order to determine the mechanism by which the AAV-BDNF treatment was working to improve function in these mice, we conducted Western Blot analysis on brain tissue from the cortex and hippocampus after 21 DPI and examined levels of BDNF isoforms as well as trkB activation. We observed that the AAV-BDNF virus does indeed elevate levels of BDNF, particularly mature BDNF, in both the cortex and the hippocampus (Figures 11A–C). In addition, we found elevated levels of p-trkB/trkB in AAV-BDNF treated Val66Met injured mice relative to control treated Val66Met injured mice in both the cortex and the hippocampus (Figures 11D,E), signifying increased activation of the mature BDNF trkB signaling pathway. Together, these results suggest that disruptions in levels of BDNF play a role in the detrimental effects seen in the Val66Met injured mice and that targeted treatment to elevate BDNF can rescue the effects of repeated mild injury in this vulnerable genotype.

## Discussion

In this study, we show that BDNF Val66Met genetic polymorphism results in worse outcomes in terms of imaging, biological correlates, and behavior relative to Val66Val genotype following repeated mild LFP injury in mice. BDNF has been shown to play an important role after injury. Early studies showed that after injury, levels of BDNF protein and mRNA are upregulated but the isoforms were not examined at that time (57–61). Our group demonstrated that after TBI protein levels of proBDNF, proNGF, p75 receptor, and sortilin co-receptor are preferentially upregulated relative to mature BDNF and its trkB receptor which may explain why there is apoptosis and neurodegeneration after injury rather than cell survival and neural regeneration (31). These results are consistent with what has been shown in the literature, that after injury *in vivo* levels of proBDNF protein and mRNA are preferentially upregulated relative to mature BDNF (31, 61, 62). We have also demonstrated that inhibiting p75 signaling or activating trkB signaling using genetic and pharmacological approaches improves cellular and behavioral response to injury (31). Other groups have shown that activating the mature BDNF trkB signaling pathways using molecules (63), neural stem cells (64), physical exercise, and acupuncture (65) can improve outcomes after TBI.

We show that Val66Met genetic polymorphism results in worse outcomes following repeated mild LFP injury. The Val66Met mice had a larger volume of inflammation by 21 DPI relative to Val66Val mice as assessed by MRI. At 1 DPI, there was also more cell death, neurodegeneration, phosphorylated tau, and activated microglia in the Val66Met injured mice compared to the Val66Val mice in the cortex and the hippocampus. By 21 DPI, the amount of cell death was reduced to sham levels while other markers sustained their elevated levels. In addition, astrogliosis became significantly elevated in the Val66Met injured mice relative to the Val66Val injured mice. Since the cortex was the primary site of injury, we found that effects were more dramatic, demonstrating the focal components of injury. We were also able to identify diffuse effects of the injury in the hippocampus in the ipsilateral hemisphere.

Taken together, these results indicate that the Val66Met injured mice respond differentially to a repeated mild injury than Val66Val injured mice starting as early as 1 day after the final injury. This is an important and novel finding since previous research on the effect of the Val66Met genetic polymorphism has been done primarily in clinical studies, with some results concluding Val66Met is the risk factor, while others concluding that Val66Val is more vulnerable. Specifically, while some clinical studies have shown that the Val66Met polymorphism results in impaired neurocognitive performance after TBI (21) and is a risk factor for TBI in combat forces (18–20), others have found that in long-term studies of combat veterans, carriers of the Val66Met polymorphism had recovery of executive function back to baseline levels while the Val66Val carriers did not (15, 17). Moreover, other studies have shown no effect at all of the polymorphism influencing outcomes after injury (26–28). Our study is the first to investigate the role of the Val66Met genetic polymorphisms on outcomes after rmTBI in a mouse model, and to find that it is the Val66Met genetic polymorphism that is the risk allele in this type of trauma. Moreover, our data is supported by previous work investigating the role of the BDNF Val66Met genetic polymorphism in stroke and spinal cord injury. For example, after stroke there is evidence that humans with the Val66Met polymorphism do worse than those with the Val66Val polymorphism (13, 13, 66, 67).

We show here that in the acute phase, Val66Met mice have more cells undergoing apoptotic cell death than their Val66Val counterparts, and apoptosis has been shown in other studies to contribute to worse outcomes (68). However, after the acute phase of injury, levels of apoptosis often return back to baseline (69, 70), while other processes have been set in motion that continue to have a pathophysiological effect. The degeneration of neurons can be seen in the acute post injury phases as well, but it can have longer lasting effects that persist up until at least 21 days after the final injury (71). In this case, the injured Val66Met mice show higher levels of neurodegeneration which may contribute to the progressive loss of function in neurons in these mice. One specific way in which neurodegeneration can occur is the hyper phosphorylation of tau. When tau is hyper phosphorylated, it will bind to other tau proteins and create aggregates. This is a disruption of normal functioning and can lead to long-term neurodegeneration. One way in which tau phosphorylation can lead to neurodegeneration is by interfering with axonal transport. When this occurs, both retrograde trophic signaling as well as the autophagy process of defective tau proteins is disrupted, leading to long-term problems in function (39, 72, 73). Our findings are in agreement with other studies that have shown that acutely after repeated mild TBI, hyper phosphorylation of tau can emerge, while later stages of tauopathies such as accumulations of hyper phosphorylated tau like neurofibrillary tangles (NFTs) are not yet seen (74). We demonstrate that injured Val66Met mice have higher levels of p-tau than their Val66Val counterparts, which highlights the potential for p-tau to contribute to the differential development of long-term outcomes after TBI.

In addition to cell death and neurodegeneration after injury, it is also common to see inflammation and activation of the neuroimmune response. Astrocytes generally work to maintain homeostasis in the brain, and are known to play an important role in the response to TBI (75). After TBI, astrocytes are activated and repair the damage from the injury. While the actions of astrocytes at baseline tend to be beneficial, after injury the prolonged activation of astrocytes can lead to inflammation and secondary injury processes. In particular, the formation of a glial scar can impair neuronal regeneration and lead to worse long term outcomes (76). We see that 1 day after the final injury, both Val66Met and Val66Val injured mice have an elevation of activated astrocytes, but by 21 days after the final injury the Val66Met injured mice have significantly more activated astrocytes than Val66Val injured mice, suggesting they may have impaired neuronal regeneration due to the formation of a glial scar. Microglia are the resident neuroimmune cells in the brain and are activated as early as 1 day after the final injury and persist until at least 21 days after the final injury in our study. Along with astrocytes, microglia are responsible for initiating the inflammatory response after injury at which point, they become activated. Similar to the role of activated astrocytes after injury, while the initial response may be protective, if the activated microglia persist over time they will contribute to secondary injury processes and worse long term outcomes (77). We have shown that as early as 1 day after the final injury, the Val66Met injured mice have elevated activated microglia relative to Val66Val injured mice, and that this difference persists until at least 21 days after the final injury, perhaps contributing to differential long-term outcomes.

Our findings that the Val66Met polymorphism is a risk allele after rmTBI is consistent with recent reports in the literature. Recent human studies have shown that the Met allele is a risk factor after a single mild TBI in the areas of attention, executive function, memory, and overall cognition (21). In a study that examined emotional symptoms after a single mild TBI, Met carriers were found to have more emotional symptoms than Val carriers (78). The Met allele has also been found to be a risk factor after a single mild-moderate TBI in cognitive language processing speed (27). Interestingly, previous studies have shown that the Met allele was actually protective in long-term executive function after focal frontal TBI in combat veterans (15, 17), perhaps indicating the importance of the SNP interaction with type of injury sustained; however, the retrospective analysis was unable to eliminate confounding factors that may have played a role. In addition to the effects of the Val66Met polymorphism on outcomes following TBI, there have also been reports of its effect after spinal cord injury. Val66Met has also been shown to be a risk factor for a worse clinical presentation in cervical spondylotic myelopathy (79), impaired spinal cord plasticity (80), and low exercise induced serum BDNF levels after spinal cord injury (81) in humans. It is thought that after stroke Val66Met might be a risk factor for poor outcomes (13, 13, 66, 67). However, that the time course of stroke recovery may be more complicated than originally thought, with the Val66Met allele shown to be the risk allele in motor ability acutely (82), and surprisingly, Val66Val allele shown to be the risk factor chronically (83). While yet another study has posited that perhaps Val66Met carriers do not have worse overall recovery but recover using different underlying brain pathways (84). Some research has suggested that the Val66Met allele may cause an altered cytokine response (85, 86). There have been no reports of the effect that the Met66Met genotype has on outcomes after TBI at the time of writing, but studies have shown that the Met66Met allele may confer more risk than the Val66Met allele in terms of anxiety, OCD, and depression (38, 87, 88). Further investigations on the effect of the Met66Met genotype on recovery after TBI in addition to the Val66Met and Val66Val groups would be beneficial to the field.

To our knowledge, this is the first report that shows the effect of the Val66Met SNP on outcomes after repeated mild TBI. Our results provide evidence that the Val66Met genetic polymorphism confers risk after repeated mild TBI. Furthermore, we have shown that the Val66Met genetic polymorphism alters the upregulation of BDNF that occurs after injury. Previously, our group as well as others, have shown that after TBI *in vivo*, levels of proBDNF protein and its p75 receptor are preferentially upregulated relative to mature BDNF and its trkB receptor (31, 61, 62). Other groups have shown that the Val66Met genetic polymorphism does not affect the relative levels of pro and mature BDNF in naïve mice, despite the fact that the SNP is located in the prodomain of the protein (32, 33). However, importantly, it does decrease intracellular trafficking of BDNF and lowers the activity-dependent secretion of BDNF which can result in functional deficits (24). Here, we have shown that after repeated mild TBI, Met carriers had less total BDNF in the cortex by 21 DPI compared to Val carriers. While we expected to see a dose dependent effect of the Met allele, we found that simply having a single Met allele was significant enough to decrease total BDNF levels. However, it is uncertain at this time whether the Met-allele associated effects are exerted at the level of BDNF secretion or the BDNF promotor. We have also demonstrated that in the hippocampus at 1 and 21 DPI, Met carriers had significantly more pro/mature BDNF compared to Val carriers. Our data therefore suggests that these alterations in BDNF levels after injury in Met carriers may contribute to the worse outcomes seen.

Given that the Val66Met genetic polymorphism appears to be a risk allele following repeated mild TBI and that altered BDNF levels may be a contributing factor, we decided to attempt to rescue the Val66Met injured mice by injecting an AAV that overexpresses wildtype BDNF in the cortex and the hippocampus after injury. Previous studies have employed delivery of BDNF through AAV in animal models as a potential treatment for various brain pathologies, such as depression (89), stroke (43, 90), Alzheimer's disease (91), and spinal cord injury (92, 93). We chose to deliver the AAV treatment immediately after the last injury, and investigated the effect of our treatment on outcomes at 21 DPI because we observed worse outcomes in cellular markers in Met carriers as well as significant differences in BDNF levels in the cortex and hippocampus at this later time point. In addition, focusing on this later time point allows time for the AAV to express sufficiently and allows us to determine the treatment's efficacy in changing longer-term outcomes, which has more translational potential.

Here, we have shown that BDNF overexpression was able to decrease levels of astrogliosis, a well-known marker for poor outcomes after injury, in Val66Met injured mice back to the levels seen in Val66Val injured mice. BDNF overexpression was also able to reduce levels of activated microglia in injured Val66Met mice to levels equivalent to injured Val66Val mice, signaling a reduced pro-inflammatory post injury phase.

In addition to the differences in cellular markers that we observed, we also investigated the effect that AAV-BDNF has on motor and cognitive behavior outcomes. We found that AAV-BDNF is able to increase spatial learning in injured Val66Met mice at 16 and 17 DPI in the Morris Water Maze Test. However, we did not find that treatment with AAV-BDNF had any effect on motor outcomes at 15 DPI or 21 DPI in the rotarod test and balance beam assay, signifying that there are potentially other factors driving the difference between injured Val66Met and injured Val66Val mice in terms of gross vestibular motor ability. These data suggest that the AAV-BDNF treatment had a more potent effect on rescuing hippocampal neurons than on sensorimotor neurons. This may be due to the fact that hippocampal neurons have a high density of trkB receptors and are therefore more responsive to our AAV-BDNF treatment (94).

Given that previous studies have shown the importance of BDNF for hippocampal-dependent processes (95), it is logical that increasing levels of BDNF in these mice improves their recovery in learning assays after injury and may be an important specialized treatment for Val-Met carriers who have lower levels of BDNF compared to their Val-Val counterparts after injury. We found that in these AAV-BDNF treated injured Val66Met mice that injection of the virus increases levels of total and mature BDNF in the cortex and hippocampus, as well as levels of p-trkB at 21 DPI, signifying that the mature BDNF pathway is more activated in AAV-BDNF treated mice relative to control treated mice. These results offer promising evidence that by manipulating the BDNF pathway, we may be able to develop targeted therapies for Met carriers who are more susceptible to poor outcomes after injury.

Since the Val66Met SNP is at the 66 amino acid position which is in the pro domain of the BDNF protein, when the prodomain is cleaved from the mature, the resultant mature BDNF protein will have no altered sequence. However, studies have shown that the genetic polymorphism in the proBDNF gene leads to altered intracellular packaging, which affects the axonal transport of BDNF and results in decreased activity dependent section of BDNF at the synapse (24, 33). Naturally, this can become an issue in disease states such as after TBI, where there is a need for an increased mature BDNF signaling in order to stimulate repair and recovery. In addition, recent work has highlighted the importance of the prodomain itself that has been cleaved off from the mature BDNF protein. Previously thought to be inert, new research has shown that it is in fact an active ligand. Recent work has shown that the pro domain itself has been shown to promote hippocampal long-term depression (LTD) both directly (96), as well as indirectly, by binding to mature BDNF with high affinity and weakening mature BDNF's ability to inhibit hippocampal LTD (97). Importantly, the 66Met substitution changes the structure of the protein, which results in changes of its function, including inhibiting hippocampal LTD *in vivo* (96) and causing acute growth cone retraction *in vitro* (98). In the hippocampus, the pro domain with the Met allele decreases Rac activity, a mediator of synaptic plasticity (98). Newer studies have shown that the 66Met prodomain is also able to disassemble dendritic spines and eliminate synapses in hippocampal neurons, leading to impaired hippocampal dependent fear extinction behavior (99). While the role of the prodomain and the effect that the 66Met substitution are still being investigated, we hypothesize that the prodomain may also play a role in differences in outcomes between Val66Val and Val66Met carriers. Given this knowledge, treatment with AAV-BDNF which supplies the 66Val form of BDNF, may be a useful treatment for other conditions that Val66Met carriers suffer from in addition to its ability to facilitate recovery after rmTBI.

In order to translate these finding into a clinical population, there must be an appreciation for the patient population. In the real world, many people take chronic anti-inflammatory drugs for other indications. Studies have shown that evidence that anti-inflammatory drugs such as non-steroidal anti-inflammatory drugs (NSAIDS) may either impede (100) or improve (101) outcomes after TBI. These issues need to be investigated further, especially in a model of rmTBI (102).

Our study lays important experimental groundwork in the investigation of the genetic underpinning of the differential response to injury seen in the TBI patient population, and highlights the rs6265 SNP and the BDNF signaling pathway as a potential mediator of these differences. However, our study was limited by several factors that future studies should attempt to incorporate. While the benefit of our study is that we could clarify the role of a single SNP, there may be interactions of the rs6265 SNP with SNPs in other genes that could play a role in determining genetic susceptibility. We also maintained strict control over the environment that the mice were in and investigated outcomes at the set timepoints of 1 and 21 DPI. Future studies should investigate the interaction of genetic susceptibility with other factors such environmental influences, longer time points post injury, and age of the subject. Given that the LFP model has diffuse effects in the brain, we should also look at the effects on white matter tracks and other areas of the brain, including the contralateral side. In addition, we used a very controlled and replicable repeated mild LFP injury method. Given that the human TBI data seems to suggest that genetic risk factors may vary based on the different types of injury sustained, future studies should investigate the role of the rs6265 in other forms of injury as well. Finally, we found that treatment with AAV-BDNF with the 66Val form can facilitate recovery after TBI, but we did not investigate the role that 66Met prodomain might be playing after injury. This would be an interesting pathway to investigate more thoroughly, potentially by analyzing signaling through the SorCS2 pathway, or analyzing structural differences between the two forms of the prodomain. There may also be value in exploring other pharmacological treatment approaches; for example, to increase BDNF signaling pathway activation by the use of trkB agonists such as 7,8 DHF. In addition, further research into the daily use of anti-inflammatory drugs by vulnerable genotypes may provide necessary insight into the effect that these drugs have on outcomes after TBI.

Taken together, this study has investigated the role of the Val66Met genetic polymorphism on cellular markers and demonstrated the role that it plays on BDNF levels and signaling after repeated mild TBI. We have explored using overexpression of BDNF as a personalized therapy for the susceptible Met carriers, and highlighted the potential usefulness of targeting BDNF signaling pathways for treatment.

## Data Availability Statement

The datasets generated for this study are available on request to the corresponding author.

## Ethics Statement

The animal study was reviewed and approved by Rutgers University Institutional Animal Care and Use Committee (IACUC).

## Author Contributions

AG, JA, and ST-V conceived and planned the experiments and supervised data collection. AG, ST, SR, and CZ carried out the experiments. DA conducted the imaging analysis. AG, ST, and CZ analyzed data. AG interpreted the results and wrote the manuscript. All authors provided critical feedback and helped to shape the manuscript.

### Conflict of Interest

The authors declare that the research was conducted in the absence of any commercial or financial relationships that could be construed as a potential conflict of interest.

## References

[1] Taylor CA Bell JM Breiding MJ Xu L. Traumatic brain injury-related emergency department visits, hospitalizations, and deaths - United States, 2007 and 2013. MMWR Surveill Summ. (2017) 66:1–16. 10.15585/mmwr.ss6609a1 28301451 PMC5829835

[2] DePalma RG Combat TBI. Chapter 2: History, epidemiology, and injury modes. In: Kobeissy FH editor. Brain Neurotrauma: Molecular, Neuropsychological, and Rehabilitation Aspects. Boca Raton, FL: Frontiers in Neuroengineering (2015). 10.1201/b18126-3 26269865

[3] Harmon KG Drezner JA Gammons M Guskiewicz KM Halstead M Herring SA. American Medical Society for Sports Medicine position statement: concussion in sport. Br J Sports Med. (2013) 47:15–26. 10.1136/bjsports-2012-091941 23243113

[4] Vasterling JJ Brailey K Proctor SP Kane R Heeren T Franz M. Neuropsychological outcomes of mild traumatic brain injury, post-traumatic stress disorder and depression in Iraq-deployed US Army soldiers. Br J Psychiatry. (2012) 201:186–92. 10.1192/bjp.bp.111.096461 22743844

[5] Koliatsos VE Xu L Chapter 47: The problem of neurodegeneration in cumulative sports concussions: emphasis on neurofibrillary tangle formation. In: Kobeissy FH editor. Brain Neurotrauma: Molecular, Neuropsychological, and Rehabilitation Aspects. Boca Raton, FL: Frontiers in Neuroengineering (2015). 10.1201/b18126-55 26269885

[6] Brown AW Moessner AM Mandrekar J Diehl NN Leibson CL Malec JF. A survey of very-long-term outcomes after traumatic brain injury among members of a population-based incident cohort. J Neurotrauma. (2011) 28:167–76. 10.1089/neu.2010.1400 21121813 PMC3064530

[7] Nicoll JA. Genetics and head injury. Neuropathol Appl Neurobiol. (1996) 22:515–7. 10.1111/j.1365-2990.1996.tb01128.x 9004240

[8] Ji Y Lu Y Yang F Shen W Tang TT Feng L. Acute and gradual increases in BDNF concentration elicit distinct signaling and functions in neurons. Nat Neurosci. (2010) 13:302–9. 10.1038/nn.2505 20173744 PMC4780419

[9] Kossel AH Cambridge SB Wagner U Bonhoeffer T. A caged Ab reveals an immediate/instructive effect of BDNF during hippocampal synaptic potentiation. Proc Natl Acad Sci USA. (2001) 98:14702–7. 10.1073/pnas.251326998 11724927 PMC64745

[10] Korte M Griesbeck O Gravel C Carroll P Staiger V Thoenen H. Virus-mediated gene transfer into hippocampal CA1 region restores long-term potentiation in brain-derived neurotrophic factor mutant mice. Proc Natl Acad Sci USA. (1996) 93:12547–52. 10.1073/pnas.93.22.12547 8901619 PMC38029

[11] Verhagen M van der Meij A van Deurzen PA Janzing JG Arias-Vasquez A Buitelaar JK. Meta-analysis of the BDNF Val66Met polymorphism in major depressive disorder: effects of gender and ethnicity. Mol Psychiatry. (2010) 15:260–71. 10.1038/mp.2008.109 18852698

[12] Soliman F Glatt CE Bath KG Levita L Jones RM Pattwell SS. A genetic variant BDNF polymorphism alters extinction learning in both mouse and human. Science. (2010) 327:863–6. 10.1126/science.1181886 20075215 PMC2829261

[13] Kim DY Quinlan EB Gramer R Cramer SC. BDNF Val66Met polymorphism is related to motor system function after stroke. Phys Ther. (2016) 96:533–9. 10.2522/ptj.20150135 26381810 PMC4817211

[14] Lim YY Hassenstab J Cruchaga C Goate A Fagan AM Benzinger TL. BDNF Val66Met moderates memory impairment, hippocampal function and tau in preclinical autosomal dominant Alzheimer's disease. Brain. (2016) 139(Pt 10):2766–77. 10.1093/brain/aww200 27521573 PMC5815565

[15] Barbey AK Colom R Paul E Forbes C Krueger F Goldman D. Preservation of general intelligence following traumatic brain injury: contributions of the Met66 brain-derived neurotrophic factor. PLoS ONE. (2014) 9:e88733. 10.1371/journal.pone.0088733 24586380 PMC3935849

[16] Failla MD Juengst SB Arenth PM Wagner AK. Preliminary associations between brain-derived neurotrophic factor, memory impairment, functional cognition, and depressive symptoms following severe TBI. Neurorehabil Neural Repair. (2016) 30:419–30. 10.1177/1545968315600525 26276123 PMC4752939

[17] Krueger F Pardini M Huey ED Raymont V Solomon J Lipsky RH. The role of the Met66 brain-derived neurotrophic factor allele in the recovery of executive functioning after combat-related traumatic brain injury. J Neurosci. (2011) 31:598–606. 10.1523/JNEUROSCI.1399-10.2011 21228168 PMC3195417

[18] Panenka WJ Gardner AJ Dretsch MN Crynen GC Crawford FC Iverson GL. Systematic review of genetic risk factors for sustaining a mild traumatic brain injury. J Neurotrauma. (2017) 34:2093–9. 10.1089/neu.2016.4833 28100103

[19] Dretsch MN Silverberg N Gardner AJ Panenka WJ Emmerich T Crynen G. Genetics and other risk factors for past concussions in active-duty soldiers. J Neurotrauma. (2017) 34:869–75. 10.1089/neu.2016.4480 27396498

[20] Dretsch MN Williams K Emmerich T Crynen G Ait-Ghezala G Chaytow H. Brain-derived neurotropic factor polymorphisms, traumatic stress, mild traumatic brain injury, and combat exposure contribute to postdeployment traumatic stress. Brain Behav. (2016) 6:e00392. 10.1002/brb3.392 27110438 PMC4834940

[21] Narayanan V Veeramuthu V Ahmad-Annuar A Ramli N Waran V Chinna K. Missense Mutation of Brain Derived Neurotrophic Factor (BDNF) alters neurocognitive performance in patients with mild traumatic brain injury: a longitudinal study. PLoS ONE. (2016) 11:e0158838. 10.1371/journal.pone.0158838 27438599 PMC4954696

[22] Liu RJ Lee FS Li XY Bambico F Duman RS Aghajanian GK. Brain-derived neurotrophic factor Val66Met allele impairs basal and ketamine-stimulated synaptogenesis in prefrontal cortex. Biol Psychiatry. (2012) 71:996–1005. 10.1016/j.biopsych.2011.09.030 22036038 PMC3290730

[23] Mallei A Baj G Ieraci A Corna S Musazzi L Lee FS. Expression and dendritic trafficking of BDNF-6 splice variant are impaired in knock-in mice carrying human BDNF Val66Met polymorphism. Int J Neuropsychopharmacol. (2015) 18:pyv069. 10.1093/ijnp/pyv069 26108221 PMC4675980

[24] Egan MF Kojima M Callicott JH Goldberg TE Kolachana BS Bertolino A. The BDNF val66met polymorphism affects activity-dependent secretion of BDNF and human memory and hippocampal function. Cell. (2003) 112:257–69. 10.1016/S0092-8674(03)00035-7 12553913

[25] Larson-Dupuis C Chamard E Falardeau V Frasnelli J Beaulieu C Poirier J. Impact of BDNF Val66Met polymorphism on olfactory functions of female concussed athletes. Brain Inj. (2015) 29:963–70. 10.3109/02699052.2015.1016452 25950261

[26] Bagnato S Minafra L Bravata V Boccagni C Sant'angelo A Castiglione A. Brain-derived neurotrophic factor (Val66Met) polymorphism does not influence recovery from a post-traumatic vegetative state: a blinded retrospective multi-centric study. J Neurotrauma. (2012) 29:2050–9. 10.1089/neu.2011.2184 22708958

[27] McAllister TW Tyler AL Flashman LA Rhodes CH McDonald BC Saykin AJ. Polymorphisms in the brain-derived neurotrophic factor gene influence memory and processing speed one month after brain injury. J Neurotrauma. (2012) 29:1111–8. 10.1089/neu.2011.1930 22188054 PMC3325555

[28] Rostami E Krueger F Zoubak S Dal Monte O Raymont V Pardini M. BDNF polymorphism predicts general intelligence after penetrating traumatic brain injury. PLoS ONE. (2011) 6:e27389. 10.1371/journal.pone.0027389 22087305 PMC3210804

[29] Dincheva I Glatt CE Lee FS. Impact of the BDNF Val66Met polymorphism on cognition: implications for behavioral genetics. Neuroscientist. (2012) 18:439–51. 10.1177/1073858411431646 22367929 PMC3387519

[30] Binder DK Scharfman HE. Brain-derived neurotrophic factor. Growth Factors. (2004) 22:123–31. 10.1080/08977190410001723308 15518235 PMC2504526

[31] Alder J Fujioka W Giarratana A Wissocki J Thakkar K Vuong P. Genetic and pharmacological intervention of the p75NTR pathway alters morphological and behavioural recovery following traumatic brain injury in mice. Brain Inj. (2016) 30:48–65. 10.3109/02699052.2015.1088963 26579945

[32] Chen ZY Bath K McEwen B Hempstead B Lee F. Impact of genetic variant BDNF (Val66Met) on brain structure and function. Novartis Found Symp. (2008) 289:180–8; discussion 8–95. 10.1002/9780470751251.ch14 18497103 PMC2735856

[33] Chen ZY Patel PD Sant G Meng CX Teng KK Hempstead BL. Variant brain-derived neurotrophic factor (BDNF) (Met66) alters the intracellular trafficking and activity-dependent secretion of wild-type BDNF in neurosecretory cells and cortical neurons. J Neurosci. (2004) 24:4401–11. 10.1523/JNEUROSCI.0348-04.2004 15128854 PMC6729450

[34] Carlino D Leone E Di Cola F Baj G Marin R Dinelli G. Low serum truncated-BDNF isoform correlates with higher cognitive impairment in schizophrenia. J Psychiatric Res. (2011) 45:273–9. 10.1016/j.jpsychires.2010.06.012 20630543

[35] Alder J Fujioka W Lifshitz J Crockett DP Thakker-Varia S Lateral fluid percussion: model of traumatic brain injury in mice. J Visual Exp. (2011) 54:3063 10.3791/3063 PMC3217637 21876530

[36] Thompson HJ Lifshitz J Marklund N Grady MS Graham DI Hovda DA. Lateral fluid percussion brain injury: a 15-year review and evaluation. J Neurotrauma. (2005) 22:42–75. 10.1089/neu.2005.22.42 15665602

[37] Xiong Y Mahmood A Chopp M. Animal models of traumatic brain injury. Nat Rev Neurosci. (2013) 14:128–42. 10.1038/nrn3407 23329160 PMC3951995

[38] Chen ZY Jing D Bath KG Ieraci A Khan T Siao CJ. Genetic variant BDNF (Val66Met) polymorphism alters anxiety-related behavior. Science. (2006) 314:140–3. 10.1126/science.1129663 17023662 PMC1880880

[39] Mannix R Meehan WP Mandeville J Grant PE Gray T Berglass J. Clinical correlates in an experimental model of repetitive mild brain injury. Ann Neurol. (2013) 74:65–75. 10.1002/ana.23858 23922306 PMC6312716

[40] Mannix R Berglass J Berkner J Moleus P Qiu J Andrews N. Chronic gliosis and behavioral deficits in mice following repetitive mild traumatic brain injury. J Neurosurg. (2014) 121:1342–50. 10.3171/2014.7.JNS14272 25267088 PMC5660896

[41] Mouzon B Chaytow H Crynen G Bachmeier C Stewart J Mullan M. Repetitive mild traumatic brain injury in a mouse model produces learning and memory deficits accompanied by histological changes. J Neurotrauma. (2012) 29:2761–73. 10.1089/neu.2012.2498 22900595 PMC13588329

[42] Bolton AN Saatman KE. Regional neurodegeneration and gliosis are amplified by mild traumatic brain injury repeated at 24-hour intervals. J Neuropathol Exp Neurol. (2014) 73:933–47. 10.1097/NEN.0000000000000115 25232942 PMC4170569

[43] Yu SJ Tseng KY Shen H Harvey BK Airavaara M Wang Y. Local administration of AAV-BDNF to subventricular zone induces functional recovery in stroke rats. PLoS ONE. (2013) 8:e81750. 10.1371/journal.pone.0081750 24312581 PMC3847037

[44] Henry RA Hughes SM Connor B. AAV-mediated delivery of BDNF augments neurogenesis in the normal and quinolinic acid-lesioned adult rat brain. Eur J Neurosci. (2007) 25:3513–25. 10.1111/j.1460-9568.2007.05625.x 17610571

[45] Aungst SL Kabadi SV Thompson SM Stoica BA Faden AI. Repeated mild traumatic brain injury causes chronic neuroinflammation, changes in hippocampal synaptic plasticity, and associated cognitive deficits. J Cereb Blood Flow Metab. (2014) 34:1223–32. 10.1038/jcbfm.2014.75 24756076 PMC4083389

[46] Loane DJ Kumar A Stoica BA Cabatbat R Faden AI. Progressive neurodegeneration after experimental brain trauma: association with chronic microglial activation. J Neuropathol Exp Neurol. (2014) 73:14–29. 10.1097/NEN.0000000000000021 24335533 PMC4267248

[47] Ziebell JM Ray-Jones H Lifshitz J. Nogo presence is inversely associated with shifts in cortical microglial morphology following experimental diffuse brain injury. Neuroscience. (2017) 359:209–23. 10.1016/j.neuroscience.2017.07.027 28736137 PMC5597855

[48] Beer R Franz G Srinivasan A Hayes RL Pike BR Newcomb JK. Temporal profile and cell subtype distribution of activated caspase-3 following experimental traumatic brain injury. J Neurochem. (2000) 75:1264–73. 10.1046/j.1471-4159.2000.0751264.x 10936210

[49] Schmued LC Stowers CC Scallet AC Xu L. Fluoro-Jade C results in ultra-high resolution and contrast labeling of degenerating neurons. Brain Res. (2005) 1035:24–31. 10.1016/j.brainres.2004.11.054 15713273

[50] Kondo A Shahpasand K Mannix R Qiu J Moncaster J Chen CH. Antibody against early driver of neurodegeneration cis P-tau blocks brain injury and tauopathy. Nature. (2015) 523:431–6. 10.1038/nature14658 26176913 PMC4718588

[51] Shively SB Edgerton SL Iacono D Purohit DP Qu BX Haroutunian V. Localized cortical chronic traumatic encephalopathy pathology after single, severe axonal injury in human brain. Acta Neuropathol. (2017) 133:353–66. 10.1007/s00401-016-1649-7 27885490 PMC5325841

[52] Jurynec MJ Riley CP Gupta DK Nguyen TD McKeon RJ Buck CR. TIGR is upregulated in the chronic glial scar in response to central nervous system injury and inhibits neurite outgrowth. Mol Cell Neurosci. (2003) 23:69–80. 10.1016/S1044-7431(03)00019-8 12799138

[53] Yu H Wang DD Wang Y Liu T Lee FS Chen ZY. Variant brain-derived neurotrophic factor Val66Met polymorphism alters vulnerability to stress and response to antidepressants. J Neurosci. (2012) 32:4092–101. 10.1523/JNEUROSCI.5048-11.2012 22442074 PMC3319323

[54] Ouyang W Yan Q Zhang Y Fan Z. Moderate injury in motor-sensory cortex causes behavioral deficits accompanied by electrophysiological changes in mice adulthood. PLoS ONE. (2017) 12:e0171976. 10.1371/journal.pone.0171976 28196142 PMC5308857

[55] Yin TC Voorhees JR Genova RM Davis KC Madison AM Britt JK. Acute axonal degeneration drives development of cognitive, motor, and visual deficits after blast-mediated traumatic brain injury in mice. eNeuro. (2016) 3:ENEURO.0220-16.2016. 10.1523/ENEURO.0220-16.2016 27822499 PMC5086797

[56] Sackheim AM Stockwell D Villalba N Haines L Scott CL Russell S. Traumatic brain injury impairs sensorimotor function in mice. J Surg Res. (2017) 213:100–9. 10.1016/j.jss.2017.02.016 28601302 PMC5996987

[57] Hicks RR Numan S Dhillon HS Prasad MR Seroogy KB. Alterations in BDNF and NT-3 mRNAs in rat hippocampus after experimental brain trauma. Brain Res Mol Brain Res. (1997) 48:401–6. 10.1016/S0169-328X(97)00158-7 9332737

[58] Marciano PG Eberwine JH Ragupathi R Saatman KE Meaney DF McIntosh TK. Expression profiling following traumatic brain injury: a review. Neurochem Res. (2002) 27:1147–55. 10.1023/A:1020973308941 12462413

[59] Yang K Perez-Polo JR Mu XS Yan HQ Xue JJ Iwamoto Y Increased expression of brain-derived neurotrophic factor but not neurotrophin-3 mRNA in rat brain after cortical impact injury. J Neurosci Res. (1996) 44:157–64. 10.1002/(SICI)1097-4547(19960415)44:2<157::AID-JNR8>3.0.CO;2-C 8723224

[60] Shah SA Prough DS Garcia JM DeWitt DS Hellmich HL. Molecular correlates of age-specific responses to traumatic brain injury in mice. Exp Gerontol. (2006) 41:1201–5. 10.1016/j.exger.2006.07.006 16978820

[61] Hicks RR Li C Zhang L Dhillon HS Prasad MR Seroogy KB. Alterations in BDNF and trkB mRNA levels in the cerebral cortex following experimental brain trauma in rats. J Neurotrauma. (1999) 16:501–10. 10.1089/neu.1999.16.501 10391366

[62] Rostami E Krueger F Plantman S Davidsson J Agoston D Grafman J. Alteration in BDNF and its receptors, full-length and truncated TrkB and p75(NTR) following penetrating traumatic brain injury. Brain Res. (2014) 1542:195–205. 10.1016/j.brainres.2013.10.047 24192075

[63] Marshall J Szmydynger-Chodobska J Rioult-Pedotti MS Lau K Chin AT Kotla SKR. TrkB-enhancer facilitates functional recovery after traumatic brain injury. Scient Rep. (2017) 7:10995. 10.1038/s41598-017-11316-8 28887487 PMC5591207

[64] Chen T Wu Y Wang Y Zhu J Chu H Kong L. Brain-derived neurotrophic factor increases synaptic protein levels via the MAPK/Erk signaling pathway and Nrf2/Trx axis following the transplantation of neural stem cells in a rat model of traumatic brain injury. Neurochem Res. (2017) 42:3073–83. 10.1007/s11064-017-2340-7 28780733

[65] Li X Chen C Yang X Wang J Zhao ML Sun H. Acupuncture improved neurological recovery after traumatic brain injury by activating BDNF/TrkB pathway. eCAM. (2017) 2017:8460145. 10.1155/2017/8460145 28243312 PMC5294361

[66] Kim EJ Park CH Chang WH Lee A Kim ST Shin YI. The brain-derived neurotrophic factor Val66Met polymorphism and degeneration of the corticospinal tract after stroke: a diffusion tensor imaging study. Eur J Neurol. (2015) 23:76–84. 10.1111/ene.12791 26228236

[67] Westbroek EM Pawlikowska L Lawton MT McCulloch CE Young WL Kim H. Brain-derived neurotrophic factor Val66Met polymorphism predicts worse functional outcome after surgery in patients with unruptured brain arteriovenous malformation. Stroke. (2012) 43:2255–7. 10.1161/STROKEAHA.112.663096 22773554 PMC3431194

[68] Nathoo N Narotam PK Agrawal DK Connolly CA van Dellen JR Barnett GH. Influence of apoptosis on neurological outcome following traumatic cerebral contusion. J Neurosurg. (2004) 101:233–40. 10.3171/jns.2004.101.2.0233 15309913

[69] Elmore S. Apoptosis: a review of programmed cell death. Toxicol Pathol. (2007) 35:495–516. 10.1080/01926230701320337 17562483 PMC2117903

[70] Zupanc GK Kompass KS Horschke I Ott R Schwarz H. Apoptosis after injuries in the cerebellum of adult teleost fish. Exp Neurol. (1998) 152:221–30. 10.1006/exnr.1998.6853 9710521

[71] Schmued LC Albertson C Slikker W Jr. Fluoro-Jade: a novel fluorochrome for the sensitive and reliable histochemical localization of neuronal degeneration. Brain Res. (1997) 751:37–46. 10.1016/S0006-8993(96)01387-X 9098566

[72] Noble W Hanger DP Miller CC Lovestone S. The importance of tau phosphorylation for neurodegenerative diseases. Front Neurol. (2013) 4:83. 10.3389/fneur.2013.00083 23847585 PMC3696910

[73] Lucke-Wold BP Turner RC Logsdon AF Bailes JE Huber JD Rosen CL. Linking traumatic brain injury to chronic traumatic encephalopathy: identification of potential mechanisms leading to neurofibrillary tangle development. J Neurotrauma. (2014) 31:1129–38. 10.1089/neu.2013.3303 24499307 PMC4089022

[74] Ojo JO Mouzon B Greenberg MB Bachmeier C Mullan M Crawford F. Repetitive mild traumatic brain injury augments tau pathology and glial activation in aged hTau mice. J Neuropath Exp Neurol. (2013) 72:137–51. 10.1097/NEN.0b013e3182814cdf 23334597

[75] Burda JE Bernstein AM Sofroniew MV. Astrocyte roles in traumatic brain injury. Exp Neurol. (2016) 275(Pt3):305–15. 10.1016/j.expneurol.2015.03.020 25828533 PMC4586307

[76] Fitch MT Silver J. CNS injury, glial scars, and inflammation: inhibitory extracellular matrices and regeneration failure. Exp Neurol. (2008) 209:294–301. 10.1016/j.expneurol.2007.05.014 17617407 PMC2268907

[77] Karve IP Taylor JM Crack PJ. The contribution of astrocytes and microglia to traumatic brain injury. Br J Pharm. (2016) 173:692–702. 10.1111/bph.13125 25752446 PMC4742296

[78] Wang YJ Chen KY Kuo LN Wang WC Hsu YW Wong HS. The association between BDNF Val66Met polymorphism and emotional symptoms after mild traumatic brain injury. BMC Med Genet. (2018) 19:13. 10.1186/s12881-017-0518-0 29357818 PMC5776765

[79] Abode-Iyamah KO Stoner KE Grossbach AJ Viljoen SV McHenry CL Petrie MA. Effects of brain derived neurotrophic factor Val66Met polymorphism in patients with cervical spondylotic myelopathy. J Clin Neurosci. (2016) 24:117–21. 10.1016/j.jocn.2015.07.016 26461908

[80] Lamy JC Boakye M. BDNF Val66Met polymorphism alters spinal DC stimulation-induced plasticity in humans. J Neurophysiol. (2013) 110:109–16. 10.1152/jn.00116.2013 23576701

[81] Leech KA Hornby TG. High-intensity locomotor exercise increases brain-derived neurotrophic factor in individuals with incomplete spinal cord injury. J Neurotrauma. (2017) 34:1240–8. 10.1089/neu.2016.4532 27526567 PMC5359683

[82] Qin L Kim E Ratan R Lee FS Cho S. Genetic variant of BDNF (Val66Met) polymorphism attenuates stroke-induced angiogenic responses by enhancing anti-angiogenic mediator CD36 expression. J Neurosci. (2011) 31:775–83. 10.1523/JNEUROSCI.4547-10.2011 21228186 PMC3308129

[83] Qin L Jing D Parauda S Carmel J Ratan RR Lee FS. An adaptive role for BDNF Val66Met polymorphism in motor recovery in chronic stroke. J Neurosci. (2014) 34:2493–502. 10.1523/JNEUROSCI.4140-13.2014 24523540 PMC3921423

[84] Di Pino G Pellegrino G Capone F Assenza G Florio L Falato E. Val66Met BDNF polymorphism implies a different way to recover from stroke rather than a worse overall recoverability. Neuroreh Neural Repair. (2016) 30:3–8. 10.1177/1545968315583721 25896987

[85] Lotrich F. Inflammatory cytokines, growth factors, and depression. Curr Pharm Des. (2012) 18:5920–35. 10.2174/138161212803523680 22681170

[86] Notaras M Du X Gogos J van den Buuse M Hill RA. The BDNF Val66Met polymorphism regulates glucocorticoid-induced corticohippocampal remodeling and behavioral despair. Transl Psychiatry. (2017) 7:e1233. 10.1038/tp.2017.205 28926000 PMC5639248

[87] Montag C Basten U Stelzel C Fiebach CJ Reuter M. The BDNF Val66Met polymorphism and anxiety: support for animal knock-in studies from a genetic association study in humans. Psychiatry Res. (2010) 179:86–90. 10.1016/j.psychres.2008.08.005 20478625

[88] Katerberg H Lochner C Cath DC de Jonge P Bochdanovits Z Moolman-Smook JC. The role of the brain-derived neurotrophic factor (BDNF) val66met variant in the phenotypic expression of obsessive-compulsive disorder (OCD). Am J Med Genet B Neuropsych Genet. (2009) 150b:1050–62. 10.1002/ajmg.b.30930 19219856

[89] Ma XC Liu P Zhang XL Jiang WH Jia M Wang CX. Intranasal delivery of recombinant AAV containing BDNF fused with HA2TAT: a potential promising therapy strategy for major depressive disorder. Scient Rep. (2016) 6:22404. 10.1038/srep22404 26935651 PMC4776097

[90] Mitroshina capital Ie C Mishchenko TA Usenko AV Epifanova EA Yarkov RS Gavrish MS. AAV-Syn-BDNF-EGFP virus construct exerts neuroprotective action on the hippocampal neural network during hypoxia *in vitro*. Int J Mol Sci. (2018) 19:2297. 10.3390/ijms19082295 30081596 PMC6121472

[91] Jiao SS Shen LL Zhu C Bu XL Liu YH Liu CH. Brain-derived neurotrophic factor protects against tau-related neurodegeneration of Alzheimer's disease. Transl Psychiatry. (2016) 6:e907. 10.1038/tp.2016.186 27701410 PMC5315549

[92] Ziemlinska E Kugler S Schachner M Wewior I Czarkowska-Bauch J Skup M. Overexpression of BDNF increases excitability of the lumbar spinal network and leads to robust early locomotor recovery in completely spinalized rats. PLoS ONE. (2014) 9:e88833. 10.1371/journal.pone.0088833 24551172 PMC3925164

[93] Eaton MJ Blits B Ruitenberg MJ Verhaagen J Oudega M. Amelioration of chronic neuropathic pain after partial nerve injury by adeno-associated viral (AAV) vector-mediated over-expression of BDNF in the rat spinal cord. Gene Therapy. (2002) 9:1387–95. 10.1038/sj.gt.3301814 12365004

[94] Spencer-Segal JL Waters EM Bath KG Chao MV McEwen BS Milner TA. Distribution of phosphorylated TrkB receptor in the mouse hippocampal formation depends on sex and estrous cycle stage. J Neurosci. (2011) 31:6780–90. 10.1523/JNEUROSCI.0910-11.2011 21543608 PMC3108038

[95] Leal G Bramham CR Duarte CB. BDNF and hippocampal synaptic plasticity. Vitamins Horm. (2017) 104:153–95. 10.1016/bs.vh.2016.10.004 28215294

[96] Mizui T Ishikawa Y Kumanogoh H Lume M Matsumoto T Hara T. BDNF pro-peptide actions facilitate hippocampal LTD and are altered by the common BDNF polymorphism Val66Met. Proc Natl Acad Sci USA. (2015) 112:E3067–74. 10.1073/pnas.1422336112 26015580 PMC4466729

[97] Uegaki K Kumanogoh H Mizui T Hirokawa T Ishikawa Y Kojima M. BDNF binds its pro-peptide with high affinity and the common Val66Met polymorphism attenuates the interaction. Int J Mol Sci. (2017) 18:1042. 10.3390/ijms18051042 28498321 PMC5454954

[98] Anastasia A Deinhardt K Chao MV Will NE Irmady K Lee FS. Val66Met polymorphism of BDNF alters prodomain structure to induce neuronal growth cone retraction. Nat Commun. (2013) 4:2490. 10.1038/ncomms3490 24048383 PMC3820160

[99] Giza JI Kim J Meyer HC Anastasia A Dincheva I Zheng CI The BDNF Val66Met prodomain disassembles dendritic spines altering fear extinction circuitry and behavior. Neuron. (2018) 99:163–78.e6. 10.1016/j.neuron.2018.05.024 29909994 PMC6054457

[100] Browne KD Iwata A Putt ME Smith DH. Chronic ibuprofen administration worsens cognitive outcome following traumatic brain injury in rats. Exp Neurol. (2006) 201:301–7. 10.1016/j.expneurol.2006.04.008 16764859

[101] Lagraoui M Sukumar G Latoche JR Maynard SK Dalgard CL Schaefer BC. Salsalate treatment following traumatic brain injury reduces inflammation and promotes a neuroprotective and neurogenic transcriptional response with concomitant functional recovery. Brain Behav Immun. (2017) 61:96–109. 10.1016/j.bbi.2016.12.005 27939247 PMC5316369

[102] Bergold PJ. Treatment of traumatic brain injury with anti-inflammatory drugs. Exp Neurol. (2016) 275 Pt 3(Pt 3):367–80. 10.1016/j.expneurol.2015.05.024 26112314 PMC6007860

