# Supplementary material for: BDNF Val66Met Genetic Polymorphism Results in Poor Recovery Following Repeated Mild Traumatic Brain Injury in a Mouse Model and Treatment With AAV-BDNF Improves Outcomes
Source: Front Neurol. 2019 Nov 7;10:1175. doi: 10.3389/fneur.2019.01175 (PMC6854037; doi:10.3389/fneur.2019.01175)
Supplement: Supplementary file 1 [file Table_1.docx]

Supplementary Materials

**Supplemental Figure 1.** **Representative images of T2 magnetic resonance imaging scans of individual mice after 1, 7, and 21 days after rmTBI or sham procedure**. Arrow indicates site of craniectomy and injury. Scans were conducted at the Rutgers University Molecular Imaging Center with the center’s M2 Compact High-Performance MRI (1T). Scale bars = 3mm.

**Supplemental Figure 2.** **AAV-BDNF GFP injection is expressed in the cortex and hippocampus, and in glia.** (A) Representative image showing AAV-BDNF GFP expression in the cortex and hippocampus at 21 DPI at 10X magnification. Green represents virus GFP expression. (B) Representative image showing AAV-BDNF GFP expression in the cortex at 40X magnification. (C) Representative image showing AAV-BDNF GFP expression in the contralateral cortex at 10X magnification. Green represents virus GFP expression Scale bars = 100µm.

**Supplemental Figure 3. Schematic of the behavioral timeline.** The same set of BDNF mice underwent a behavioral battery paradigm lasting 27 days in total. (Abbreviations are as follows: MWM = Morris Water Maze, RR = Rotarod, BB = Balance Beam.)

**Supplemental Figure 4. Statistical data for the figures.** Statistics were carried out on the data sets that were analyzed and the significant differences between groups were noted in the individual figures. This figure provides the p-values for these differences.

**Supplemental Figure 5. Representative examples of inflammation.** A) Representative images of BDNF Val66Met LFP cortical sections at 1 DPI stained with IBA1 on the ipsilateral side to the injury at 10X and 40X magnification. B) Representative images of BDNF Val66Met LFP cortical sections at 1 DPI stained with IBA1 on the contralateral side to the injury at 10X and 40X magnification. C) Representative images of BDNF Val66Met Sham cortical sections at 1 DPI stained with IBA1 on the ipsilateral side to the injury at 10X and 40X magnification. White arrows indicate resting microglia, white arrowheads indicate activated microglia. Scale bars = 100µm.
