# Supplementary figures and images for: BDNF Val66Met Genetic Polymorphism Results in Poor Recovery Following Repeated Mild Traumatic Brain Injury in a Mouse Model and Treatment With AAV-BDNF Improves Outcomes
Source: Front Neurol. 2019 Nov 7;10:1175. doi: 10.3389/fneur.2019.01175 (PMC6854037; doi:10.3389/fneur.2019.01175)

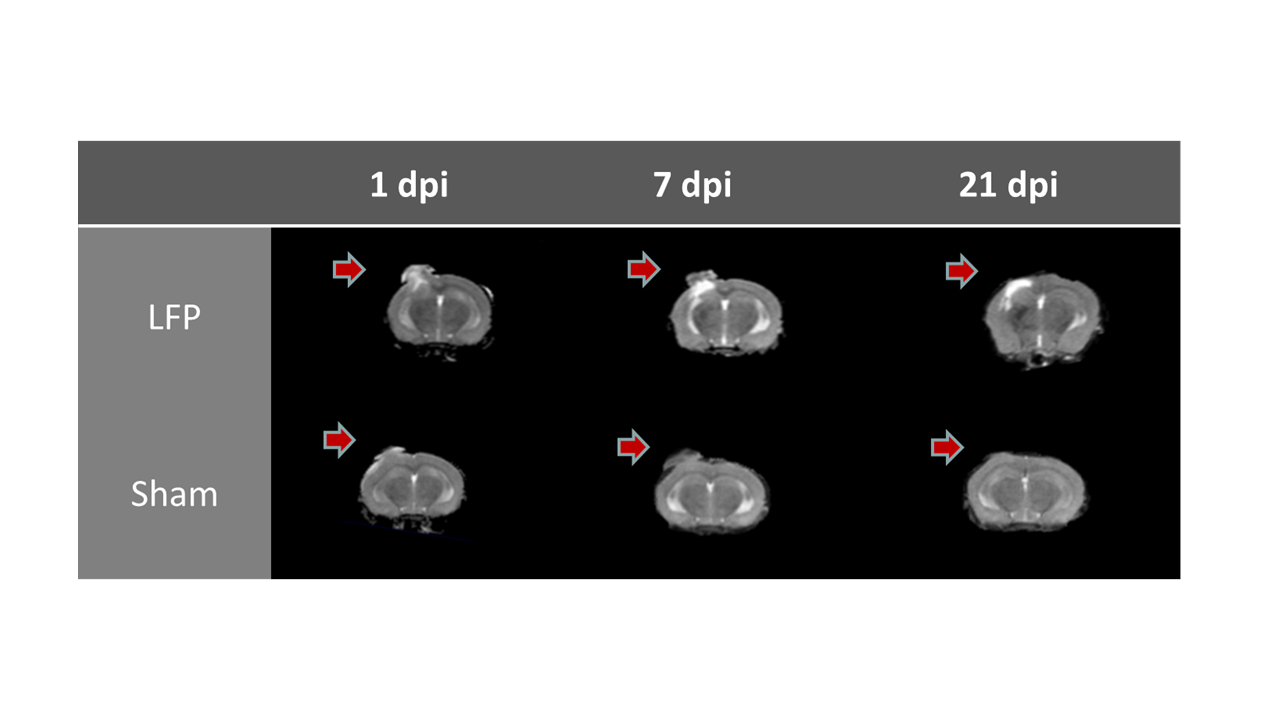

Supplement: Supplementary file 2 [file Image_1.TIF]

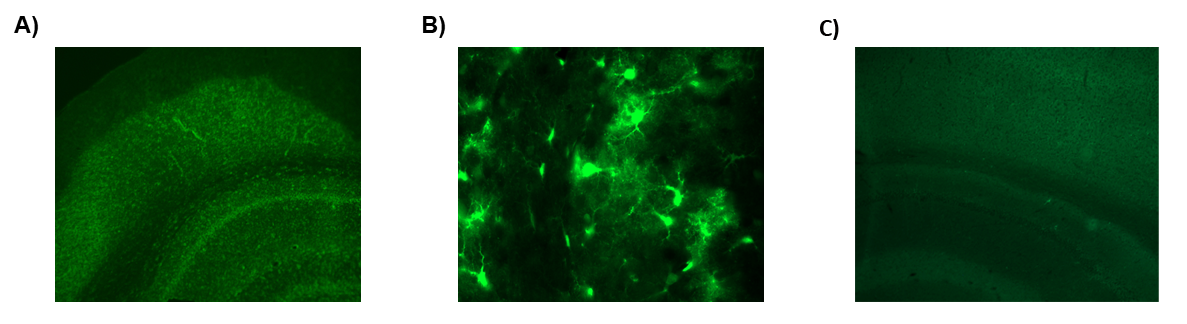

Supplement: Supplementary file 3 [file Image_2.TIF]

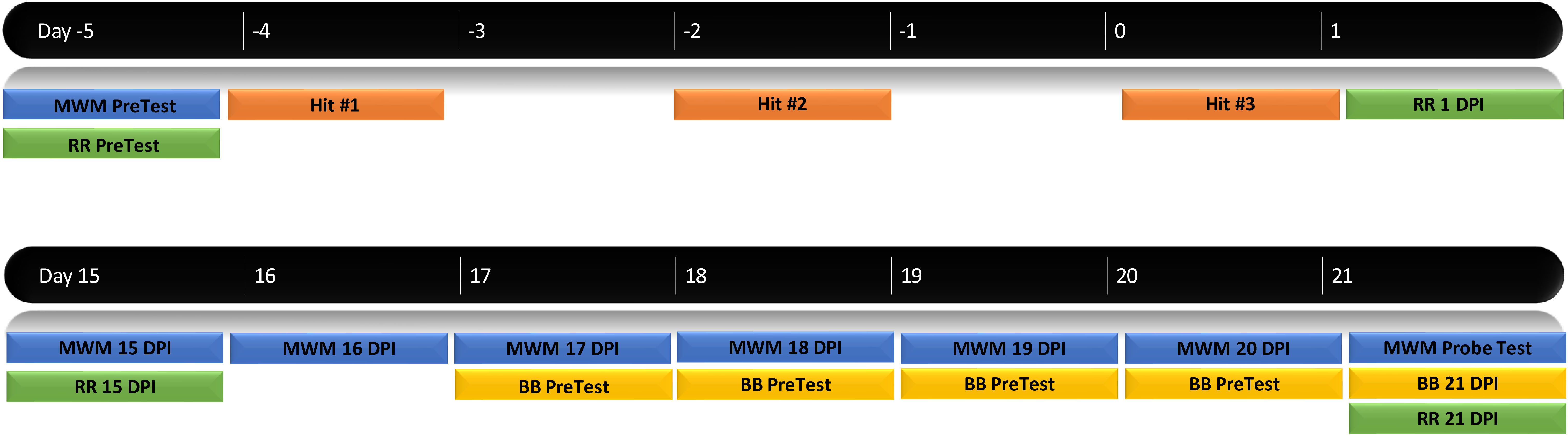

Supplement: Supplementary file 4 [file Image_3.TIF]

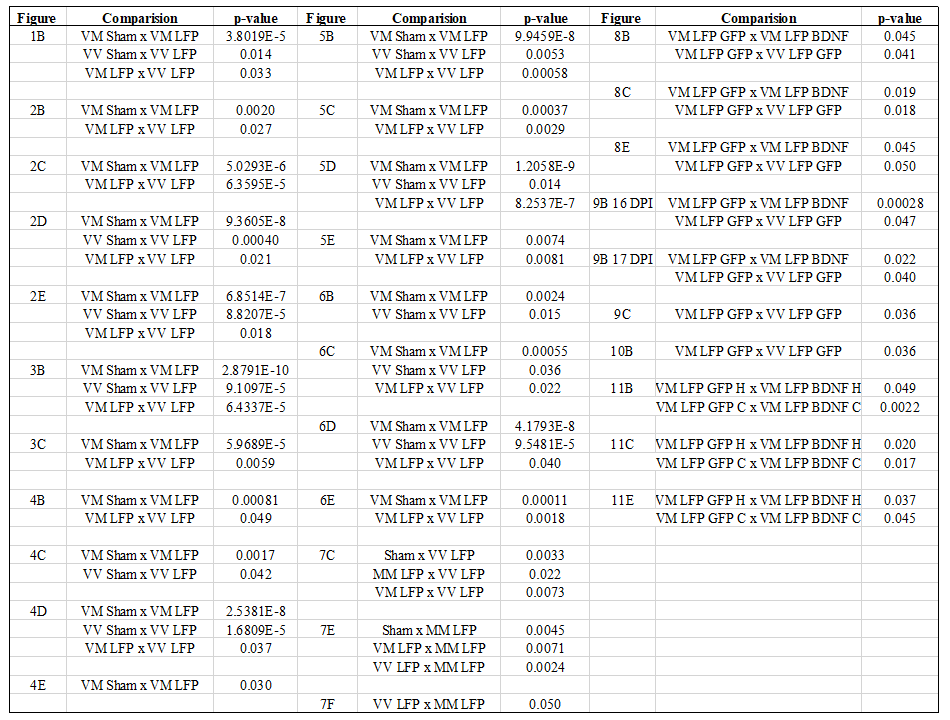

Supplement: Supplementary file 5 [file Image_4.TIF]

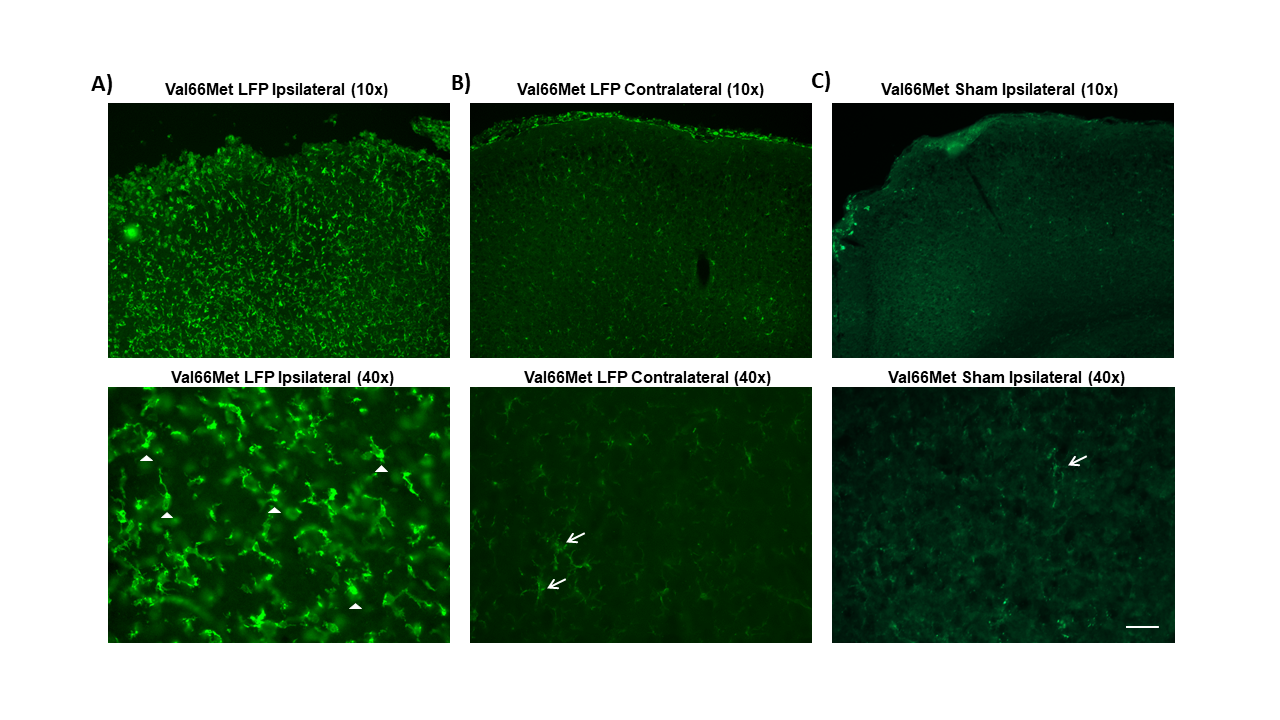

Supplement: Supplementary file 6 [file Image_5.TIF]
